# *zic-1* Expression in Planarian Neoblasts after Injury Controls Anterior Pole Regeneration

**DOI:** 10.1371/journal.pgen.1004452

**Published:** 2014-07-03

**Authors:** Constanza Vásquez-Doorman, Christian P. Petersen

**Affiliations:** 1 Department of Molecular Biosciences, Northwestern University, Evanston, Illinois, United States of America; 2 Robert Lurie Comprehensive Cancer Center, Northwestern University, Evanston, Illinois, United States of America; University of Oxford, United Kingdom

## Abstract

Mechanisms that enable injury responses to prompt regenerative outgrowth are not well understood. Planarians can regenerate essentially any tissue removed by wounding, even after decapitation, due to robust regulation of adult pluripotent stem cells of the neoblast population. Formation of pole signaling centers involving Wnt inhibitors or Wnt ligands promotes head or tail regeneration, respectively, and this process requires the use of neoblasts early after injury. We used expression profiling of purified neoblasts to identify factors needed for anterior pole formation. Using this approach, we identified *zic-1*, a Zic-family transcription factor, as transcriptionally activated in a subpopulation of neoblasts near wound sites early in head regeneration. As head regeneration proceeds, the Wnt inhibitor *notum* becomes expressed in the newly forming anterior pole in *zic-1*-expressing cells descended from neoblasts. Inhibition of *zic-1* by RNAi resulted in a failure to express *notum* at the anterior pole and to regenerate a head, but did not affect tail regeneration. Both injury and canonical Wnt signaling inhibition are required for *zic-1* expression, and double-RNAi experiments suggest *zic-1* inhibits Wnt signaling to allow head regeneration. Analysis of neoblast fate determinants revealed that *zic-1* controls specification of *notum*-expressing cells from *foxD*-expressing neoblasts to form the anterior pole, which organizes subsequent outgrowth. Specialized differentiation programs may in general underlie injury-dependent formation of tissue organizing centers used for regenerative outgrowth.

## Author Summary

Some animals are capable of regenerating organs damaged or removed by injury, and this ability likely requires precise control of secreted proteins that promote growth. Planarians are flatworms that can regenerate any missing tissues by regulating the activity of adult stem cells that can produce any specialized cell type. We identify the *zic-1* gene as activated in planarian stem cells by injury and needed for head regeneration after decapitation. This gene's product likely acts as a transcription factor to produce cells that secrete a growth-promoting protein, NOTUM, at the tip of the regenerating tissue outgrowth to organize and enable head regeneration. These results suggest that regeneration requires specialized uses of stem cell descendants to orchestrate new tissue production following injury.

## Introduction

Regeneration is widespread in animals but still poorly understood. In animals with this ability, injury responses and positional cues likely interact to signal the production of missing structures. Across vertebrates and invertebrates, regeneration can involve either stem cells of high potency or cells with more limited potential [1]. Therefore, rather than the presence or absence of a single regenerative cell type, patterning systems that directly or indirectly control such cells may instead be the critical determinate for regenerative abilities. Injuries can alter tissue variously, so regenerating animals must have robust cell signaling mechanisms that enable appropriate restoration of structures damaged or lost by wounding. An important unresolved issue in understanding regeneration mechanisms is whether stem cells function only in production of differentiated cell types that comprise fully regenerated structures, or whether they could have additional functions in directing outgrowth by controlling tissue patterning.

Planarians can regenerate nearly any tissue damaged by injury [2], and the processes of head or tail regeneration serve as simple models for study of mechanisms that relate wounding to stem cell activation and growth signaling. Planarians rely on adult pluripotent stem cells of the neoblast population for producing all differentiated cell types needed for whole-body regeneration and for viability through ongoing tissue homeostasis [3]. Neoblasts express the PIWI homolog *smedwi-1* [4] and are the only known proliferating cells in planarians [5], so FACS isolation of G2/S/M cells labeled with DNA-binding vital dyes can purify this cell population [4], [6]. Neoblasts respond to injury through changes in proliferation [7], localization [8] and gene expression [9]. RNAi and small molecule treatments have implicated several signaling pathways and processes in head and tail regeneration that therefore directly or indirectly control the function of neoblasts or their differentiating progeny, including Wnt [10]–[18], BMP [19]–[23], Activin [24], [25], Hedgehog [15], [26], FGF, and calcium signaling [27], as well as communication through gap-junctions [28], [29] and bioelectric signaling [30], [31].

Neoblast-dependent processes likely provide input into the control of canonical Wnt signaling that directs head and tail regeneration and polarized responses on the anteroposterior (A/P) axis [10]–[12]. *wnt1* is required for tail regeneration and is activated early by 6 hours after injury [13], [32] in body-wall muscle cells [33] in a neoblast-independent manner. Subsequently, by 48–72 hours, in fragments regenerating a new tail, *wnt1* becomes expressed in a focus of cells at the posterior pole in a neoblast-dependent manner. By contrast, *notum* encodes a secreted hydrolase that can inhibit Wnt signaling in planarians [14], *Drosophila* [34], [35], and zebrafish [36], and is required for head regeneration in planarians [14]. Injury activates *notum* expression preferentially at anterior-facing injury sites within body-wall muscle cells [33] with kinetics similar to wound-induced *wnt1* expression. Subsequently, by 48–72 hours, in fragments regenerating a new head, *notum* is expressed in a focus of cells at the newly forming anterior pole. Temporal RNAi experiments found that *wnt1* and *notum* are required during tail and head regeneration [13], [14], indicating the importance of these expression behaviors for their growth promoting functions.

The involvement of additional genes in head and tail regeneration also points to the importance of neoblast-dependent processes in pole formation. *pbx* is a TALE class homeodomain transcription factor expressed broadly, including within neoblasts, and is required for head and tail regeneration as well as pole expression, but not wound-induced expression, of *wnt1* and *notum* [37], [38]. Tail regeneration and expression of *wnt1* in the tail requires the transcription factor *pitx* [39], [40]. Additionally, the forkhead transcription factor *foxD* is activated early by 6 hours in body-wall muscle cells at injury sites that disrupt the midline, including anterior and posterior amputation sites, is subsequently expressed in neoblasts and the anterior pole, and is needed for *notum* anterior pole expression and head regeneration [41]. Therefore, these studies indicate that pole formation is either a consequence of neoblast-dependent growth or alternatively might require specialized differentiation activities of neoblasts.

We used transcriptional profiling to identify a Zic transcription factor, *Smed-zic-1*, as a likely candidate for control of neoblasts to produce an anterior pole in head regeneration. Zinc fingers of the cerebellum (Zic) proteins, homologous to *Drosophila odd-paired*, are conserved in animals, can act as transcription factors, and participate in axis formation, neurogenesis, and mesoderm formation [42]–[49]. However, pleiotropy and redundancy have complicated the identification of specific shared functions for vertebrate Zic genes, and loss-of-function studies have not yet pointed to their common use in only a single signaling pathway [50]. Additionally, functions for Zic proteins in injury responses have not been described. The injury-dependent program of adult organ formation in planarians provides a simple system to identify and clarify highly conserved, and therefore central, *zic* gene functions used in stem cell control, tissue patterning and growth. Our studies indicate that Zic proteins can participate in organ regeneration through stem cell-dependent production of signaling centers that direct subsequent tissue outgrowth.

## Results

### Identification of *zic-1* as induced in neoblasts early in head regeneration

**Figure 1:**
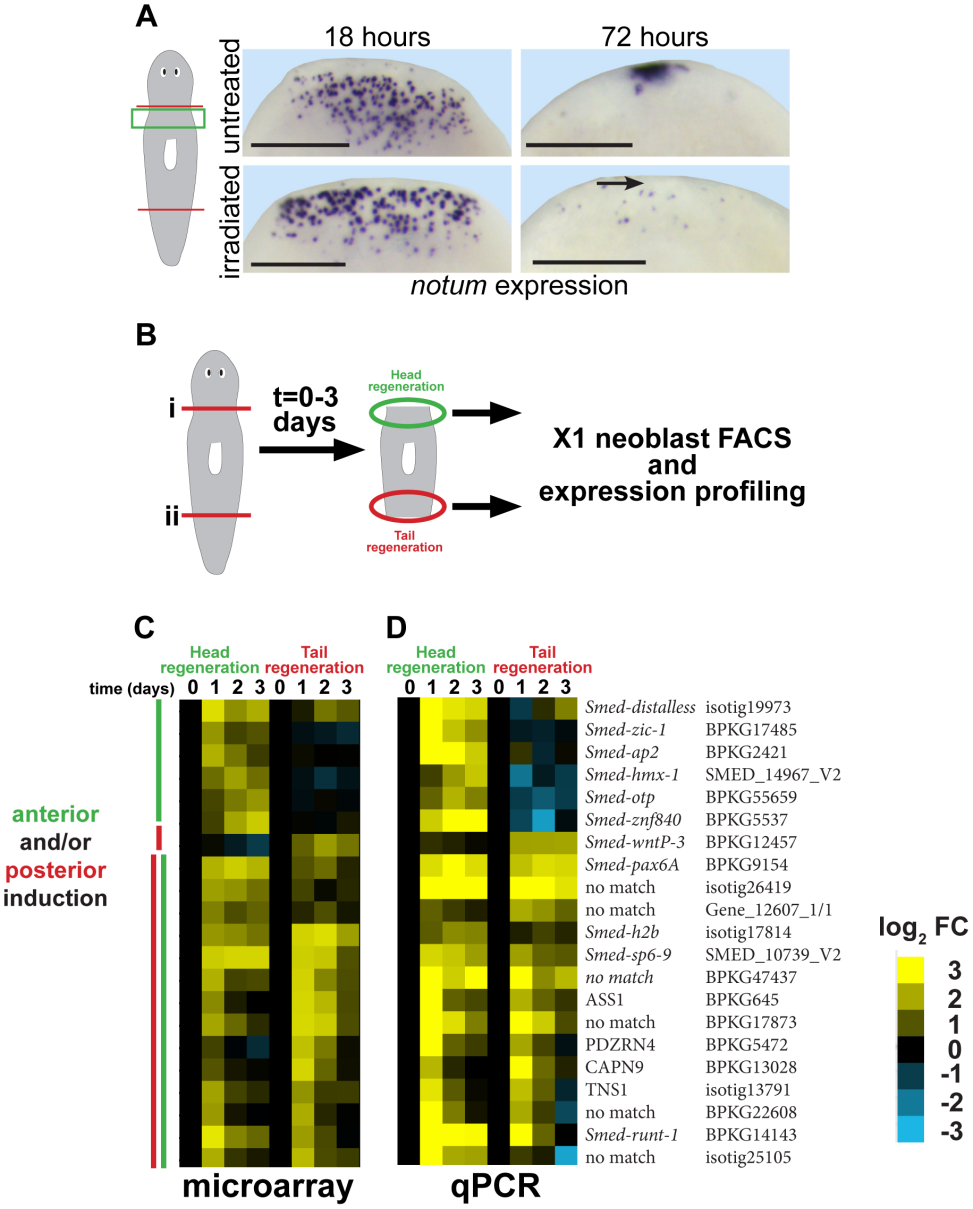
Strategy for identifying genes required for neoblast-dependent formation of the anterior pole. (A) in situ hybridizations to detect expression of *notum* after amputation in untreated animals or animals exposed to gamma irradiation (6000 Rads) 48 hours prior to surgery. Late (72 hours) expression of *notum* at the anterior pole is irradiation sensitive (arrow), but not early (18 hours) expression of *notum* in disperse cells near the wound site. Cartoon shows surgery (red lines) and enlarged region (green box). Bars, 300 microns. (B) Cartoon showing strategy to identify genes expressed differentially within neoblasts in head and tail regeneration. Animals were amputated transversely at either planes (i) or (ii), and X1 neoblasts purified by FACS from macerated tissue fragments from regions near injury sites engaged in either head or tail regeneration in a time series. Expression profiling was performed to identify genes whose expression significantly changed at 1, 2, or 3 days after amputation as compared to neoblasts isolated from regionally matched non-regenerating tissue. (C) Heat map showing only genes upregulated as detected by microarray (yellow, log_2_ fold-change (FC), chosen with false-discovery rate <10%) in head and/or tail regeneration. (D) qPCR experiment showing expression of 21/24 candidate genes upregulated in neoblasts due to regeneration whose expression behavior was confirmed upregulated. (C–D) Heat maps show microarray and qPCR data for genes ordered using hierarchical clustering with both microarray and qPCR expression values as inputs and broadly categorize into those upregulated primarily in anterior or posterior regeneration, or both (yellow, log_2_ fold-change versus 0 hour timepoint for each anterior or posterior series). We further analyzed a subset of genes that reproducibly increased in expression specific to anterior regeneration.

To identify genes involved in putative stem cell-dependent patterning processes, we first examined neoblast requirements for injury-induced expression of *notum*, a Wnt inhibitor required for head regeneration [14]. In head regeneration, *notum* expression is activated in a number of cells near the anterior-facing amputation site by 18 hours and subsequently by 48–72 hours its expression occurs prominently at the regenerating anterior pole. Animals treated with lethal doses of gamma irradiation were depleted of *smedwi-1*-positive neoblasts, as described previously [4], and succeeded in expressing *notum* at 18 hours but not at the anterior pole by 72 hours (Fig. 1A, Fig. S1). These results confirm previous observations that anterior pole formation is neoblast-dependent [41].

We reasoned that genes important for a neoblast-dependent step in anterior pole formation might be expressed in neoblasts specifically during head regeneration. To identify such genes, we used Hoechst staining and FACS to isolate X1 neoblasts (G2/S/M cells) from tissues near anterior- and posterior-facing wound sites over three days of regeneration and transcriptionally profiled these cells using custom microarrays (Agilent) performed in biological triplicates (Fig. 1B, Fig. S2A). 66 genes had expression changes predicted with a false discovery rate of 10% (Benjamini-Hochberg method for correction of multiple hypothesis testing, see Methods), of which 24 were detected as upregulated in neoblasts during regeneration with various kinetics and specificities (Fig. 1C, Fig. S2B, Table S1). We used qPCR to validate the expression behavior of these genes. Expression upregulation was confirmed for 21 of these genes (Fig. 1D), and with broadly similar kinetics as measured by microarray. Hierarchical clustering classified genes as having induction broadly polarized to anterior regeneration (*distalless, zic-1, ap2, hmx-1, otp, znf840*), posterior regeneration (*wntP-3)*, or that occurred in both anterior and posterior regeneration in either analysis. Nine of the 21 confirmed injury-induced genes encoded predicted transcription factors with six previously identified as expressed in neoblast subpopulations (*runt-1*, *hmx-1*, *ap-2*, *pax-6*, *dlx-3*, and *sp6-9)* [9], [51], [52] and three not yet identified as neoblast-expressed (*zic-1*, *znf840*, and *otp*). Of these nine genes, expression profiles from the microarray indicated four were induced in early head regeneration (24 hours) much greater than in tail regeneration: *zic-1*, *distalless (dlx)*, *ap-2*, and *hmx-1*. *dlx*, *ap2*, and *hmx-1* have reported expression patterns and functions related to regeneration of eye cells and specific neuronal subpopulations rather than pole regionalized expression or functions in head formation [9], [51], [52]. Therefore, we focused subsequent analysis on *zic-1*. A previous phylogenetic study identified this gene as one of two planarian Zic family members (previously designated as *zicA* and *zicB*), with representatives in all animals [45]. We renamed the *zicA* gene *Smed-zic-1* (hereafter referred to as *zic-1*) in keeping with the *Schmidtea mediterranea* gene nomenclature guidelines [53].

**Figure 2:**
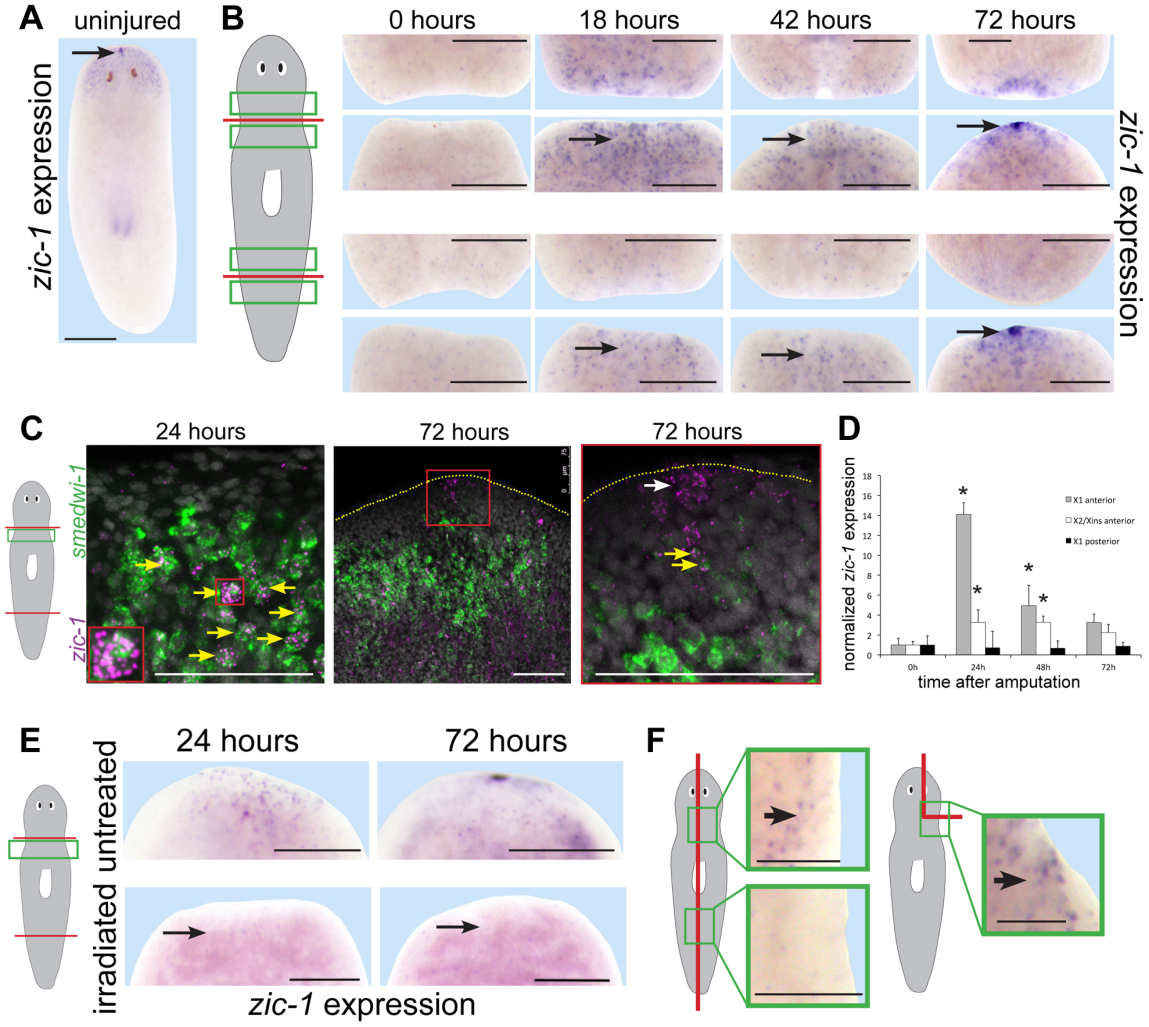
Injury induces expression of *zic-1* in neoblasts and in the regenerating anterior pole. (A–B) in situ hybridizations to detect expression of *zic-1* in uninjured or regenerating animals as indicated. (A) *zic-1* was expressed in the head region and head tip in uninjured animals (arrow). (B) *zic-1* was expressed preferentially near anterior-facing versus posterior-facing amputation sites by 18 hours and is expressed at the regenerating anterior pole by 72 hours after surgery (arrows). (C) Double fluorescence in situ hybridizations (FISH) to detect co-expression of *zic-1* (magenta) with *smedwi-1* (green) mRNA in tissue regions near anterior-facing amputation sites. Hoechst staining shows nuclei (gray). By 24 hours after injury, numbers of *zic-1+/smedwi-*1+ cells increased substantially (13/29 of *zic-1+* cells were *smedwi-1*+, n = 3 animals at 0 hours to 506/530 of *zic-1+* cells were *smedwi-1*+, n = 4 animals at 24 hours) and by 72 hours *zic-1* was expressed at the anterior pole in non-neoblast cells lacking *smedwi-1* mRNA expression (10/10 cells, n = 3 animals). Arrows indicate cells co-expressing (yellow) and not singly-expressing the two genes (white). Red box indicates enlarged region of a *zic-1+/smedwi-1+* cell (24 hours, inset) or anterior pole region (72 hours, right). Yellow dotted line shows anterior edge (72 hours). (D) qPCR to measure *zic-1* expression versus *gapdh* in FACS-sorted X1 or X2/Xins cells purified as in Fig. S2A from tissue near anterior or posterior-facing injury sites as in Fig. 1B in a time series relative to expression at 5 minutes post surgery. Error bars are standard deviations. (E) Injury-induced *zic-1* expression was eliminated in animals lethally irradiated with 6000 Rads immediately prior to amputation (arrows). (F) Early wound-induced expression of *zic-1* occurred independently of anterior removal or midline disruption. Left, laterally amputated animals activated *zic-1* expression near the injury site preferentially in animal anterior versus posterior (11/12 animals probed). Right, asymmetric anterior wedge removed tissue without midline or anterior pole disruption and resulted in expression of *zic-1* by 18 hours after surgery (6/7 animals probed). Unless otherwise noted, all panels represent at least 4 of 5 animals probed. Cartoons show surgeries and enlarged regions. Anterior, top. Bars, 250 microns (A, B, E) and 75 microns (C, F).

We first used in situ hybridizations to confirm *zic-1's* injury-induced expression behavior. In uninjured animals, *zic-1* was expressed in the head region and at the anterior pole (Fig. 2A). In animal fragments fixed during regeneration of a head and/or a tail, *zic-1* was expressed near anterior-facing injury sites by 18 hours (Fig. 2B). *zic-1* was expressed preferentially at anterior- versus posterior-facing amputation sites made at similar axial positions, although it was initially expressed more strongly at injury sites derived from the anterior (prepharyngeal) versus the posterior (postpharyngeal) of the amputated animal. By 42 hours, *zic-1* expression in posterior-facing wound sites was reduced but expression at anterior-facing sites persisted, and by 72 hours *zic-1* was expressed strongly in the anterior pole and in surrounding regions. Injury induced *zic-1* expression was not exclusive to the anterior, as expression at posterior-facing wound site of head fragments was observed by 18 hours and later near but not coincident with the posterior pole of regenerating head fragments by 72 hours. However, expression at anterior-facing injury sites was prominent and persistent. We conclude that *zic-1* expression is activated early following injury, marks the anterior pole, and occurs differentially in head versus tail regeneration.

We next performed experiments to verify expression of *zic-1* in neoblasts. We used double fluorescence in situ hybridization (FISH) to detect injury-induced *zic-1* expression in *smedwi-1*-positive neoblasts (Fig. 2C). In freshly amputated animals, a small number of *zic-1*-positive cells were identifiable, and some co-expressed *smedwi-1* (13/29 cells, n = 3 animals). By 24 hours of anterior regeneration, the number of *zic-1/smedwi-1* double-positive cells near the injury site greatly increased (506/530 cells, n = 4 animals). Additionally, there were a large number of *smedwi-1+* cells that lacked *zic-1* expression, suggesting that *zic-1* expression marks a neoblast subpopulation near injury sites. The entire neoblast population increases in number due to amputation by 24 hours [5], [7] but does so significantly less than the ∼10-fold increase in *zic-1+/smedwi-1+* cells we observed. Anterior pole cells expressing *zic-1* at 72 hours lacked expression of *smedwi-1* (10/10 cells, n = 3 animals). All non-neoblast cells are descended from neoblasts [3], so these results indicated that injury activates expression of *zic-1* in a subpopulation of neoblasts at anterior-facing injury sites and subsequently in neoblast descendants located at the regenerating anterior pole.

We performed two additional tests to confirm expression of *zic-1* in the neoblast population due to injury. First, we quantified *zic-1* expression in X1 cells by realtime PCR, and observed a 14-fold induction of *zic-1* expression during head regeneration by 24 hours that was specific to anterior-facing injury sites (Fig. 2D). X2/Xins cells representing G1 cells of the animal also increased their expression of *zic-1* due to amputation but to a lesser extent than did X1 cells. Second, we examined *zic-1* expression in animals exposed to lethal doses of gamma irradiation the day of head and tail amputation (Fig. 2E). This dose was sufficient to eliminate neoblasts, and abolished both early wound-induced and anterior pole expression of *zic-1* (8/8 animals). These experiments support the conclusion that *zic-1* expression is induced by injury in neoblasts.

Planarian genes induced transcriptionally by injury vary in their responsiveness to wound site orientation, extent and location [9], [13]–[15], [25], [32]. To examine requirements for injury-induced *zic-1* expression, we made symmetric lateral amputations or asymmetric wedges that removed part of the brain without pole removal or injuring through the animal midline (Fig. 2F). In both cases, *zic-1* was expressed in cells near the injury sites by 18 hours after wounding (lateral amputation, 12/12 worms had expression; wedge incision, 6/7 worms had expression) in irradiation-sensitive cells (lateral amputation, 0/8 worms had expression; wedge, 0/5 had expression). Additionally, wound-site proximal *zic-1* expression was preferentially anterior and not posterior in laterally amputated animals at 18 hours (11/12 animals assayed). These results indicate that *zic-1* activation in neoblasts occurs with anterior bias and independently of midline disruption, pole removal and head removal.

### *zic-1* is co-expressed with anterior pole genes in recently neoblast-derived cells

**Figure 3:**
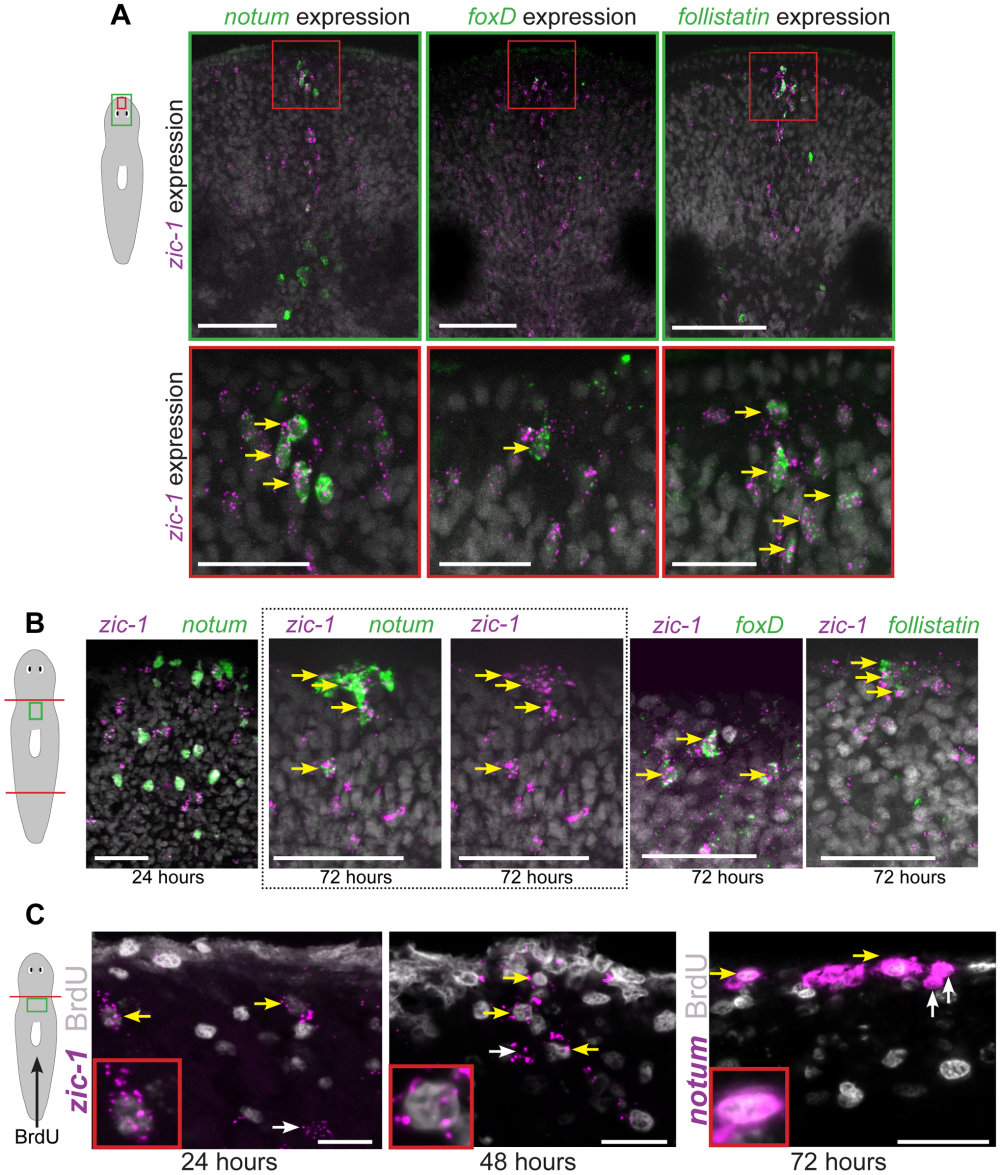
*zic-1* is co-expressed with *notum*, *foxD* and *follistatin* in neoblast descendent cells at the anterior pole. (A) Double FISH to detect expression of *zic-1* (magenta) and either *notum*, *foxD*, or *follistatin* (green) in at the anterior pole of uninjured animals with nuclei stained using Hoechst (gray). Cartoon depicts fields of view in green (top) or red (bottom) borders. (B) Double FISH as in (A) detecting expression in regenerating fragments at the indicated times after decapitation. Dotted box, contains image of anterior pole showing *zic-1* and *notum* staining (left) and corresponding image of only *zic-1* staining (right). *zic-1* is not highly co-expressed with *notum* at 24 hours (9/142 *notum+* cells were *zic-1+*, n = 3 worms), but is co-expressed with *notum* at the anterior pole by 72 hours after surgery. At 72 hours in the regenerating anterior pole, 62.1±5.3% *zic-1+* cells co-expressed *notum* (n = 3 worms), 91.7±11.8% *zic-1+* cells co-expressed *foxD* (n = 5 worms), and 56.1±28.6% *zic-1+* cells co-expressed *follistatin* (n = 6 worms). (C) Animals were injected with bromodeoxyuridine (BrdU) two days prior to decapitation, then fixed at the indicated times and probed by in situ hybridization and immunostaining to detect BrdU and either *zic-1* or *notum*. This pulse of BrdU labeled 35.2±16.6% of *zic-1+* cells at 24 hours in the parenchyma (n = 5 worms, 440 *zic-1+* cells counted), and 80.4±20% of *zic-1+* cells at the anterior pole by 48 hours (n = 5/6 worms with anterior poles, n = 49 *zic-1+* cells counted), and labeled *notum+* cells at the anterior pole by 72 hours. Yellow arrows indicate co-labeled cells, white arrows indicate single-labeled cells. Cartoons show surgeries and enlarged region. Anterior top. Bars, 20 microns (C), 30 microns(A bottom panels, B), 75 microns (A top panels).

We examined the relationship between injury-induced neoblast expression and anterior pole expression of *zic-1*. First, we tested whether *zic-1* co-expressed with other genes that together mark cells the anterior pole: *notum*, *follistatin (fst)*, and *foxD*. Double FISH experiments performed on uninjured animals detected co-expression of *zic-1* and *notum*, *zic-1* and *foxD*, and *zic-1* and *follistatin* at the anterior pole (Fig. 3A). Additionally, we performed similar experiments to analyze the relationship between these four genes during both early (18 hours) and late (48–72 hours) regeneration phases (Fig. 3B). At 18 hours, *notum* is expressed primarily in the body wall musculature [33] whereas *zic-1* is expressed primarily in neoblasts (Fig. 2C). Consistent with these observations, the majority of *notum*-expressing cells did not express *zic-1* at 24 hours of regeneration (142/151 *notum+* cells were *zic-1−*, n = 3 animals), only a small fraction of *zic-1+*/*notum+* cells were identifiable (9/151 *notum+* cells were *zic-1+*, n = 3 animals). Subsequently, at 72 hours, *notum* and *zic-1* were co-expressed in many cells at the regenerating anterior pole (34/39 *notum+* cells were *zic-1+*, n = 7 animals). Similarly, at early times in regeneration (6–18 hours), *foxD* and *follistatin* are primarily expressed in *collagen+* cells of the body wall musculature near injury sites that disrupt the midline, and at generic injury types, respectively [25], [33], [41]. By 72 hours, however, both genes were expressed in cells of the anterior pole that co-express *zic-1* (32/36 *zic-1+* cells were *foxD+*, n = 5 animals; 36/69 *zic-1+* cells were *fst+*, n = 6 animals). Therefore, *zic-1* and *notum*, *foxD*, and *follistatin* are not co-expressed early after head amputation, but later at the regenerating anterior pole.

We further used multiplex histology and labeling experiments to characterize the cell composition of the regenerating anterior pole using *notum* as a marker. Some 48-hour anterior pole cells expressing *notum* also expressed SMEDWI-1 protein (86.5±8.3% *notum+* cells co-expressed SMEDWI-1, n = 5 animals) (Fig. S3), suggesting some pole cells are recently differentiated from *smedwi-1+* neoblasts [54]. *notum+* anterior pole cells did not express *prog-1*, which marks a postmitotic population of neoblast descendants [55]. A fraction of *notum+* cells at the anterior pole of 72-hour regenerating animals expressed *collagen* (17.3±16.8% *notum+* cells co-expressed *collagen*, n = 5 animals), which marks body-wall muscle cells that express secreted regulators of regeneration [33]. Together, these results suggest that anterior pole cells include cells of the body-wall musculature formed by neoblast differentiation during regeneration.

To further examine the hypothesis that cells of the regenerating anterior pole are descended recently from neoblasts, we performed bromodeoxyuridine (BrdU) labeling experiments. As neoblasts are believed to be the only proliferating cell population in planarians, BrdU first marks neoblasts and subsequently their differentiating progeny and differentiated cell types [52], [55]. We administered a pulse of BrdU to label neoblasts two days prior to head amputation, fixed animals in a time series and detected *zic-1* mRNA expression by FISH and BrdU incorporation by immunostaining (Fig. 3C). BrdU+*zic-1+* cells were observed at 24 hours after amputation in the parenchyma near the injury site (35.2±16.6% of *zic-1+* cells were BrdU+, n = 6 worms), and at 48 hours near the anterior pole (80.4±20% of *zic-1+* cells were BrdU+, n = 5 worms). Furthermore, we detected BrdU+*notum*+ cells at the anterior pole of 72-hour regenerating fragments. Therefore, these results indicate that the anterior pole contains cells recently differentiated from neoblasts and are consistent with the hypothesis that some *zic-1+* neoblasts form *zic-1+* anterior pole cells.

### *zic-1* is needed for head regeneration and production of an anterior signaling center

**Figure 4:**
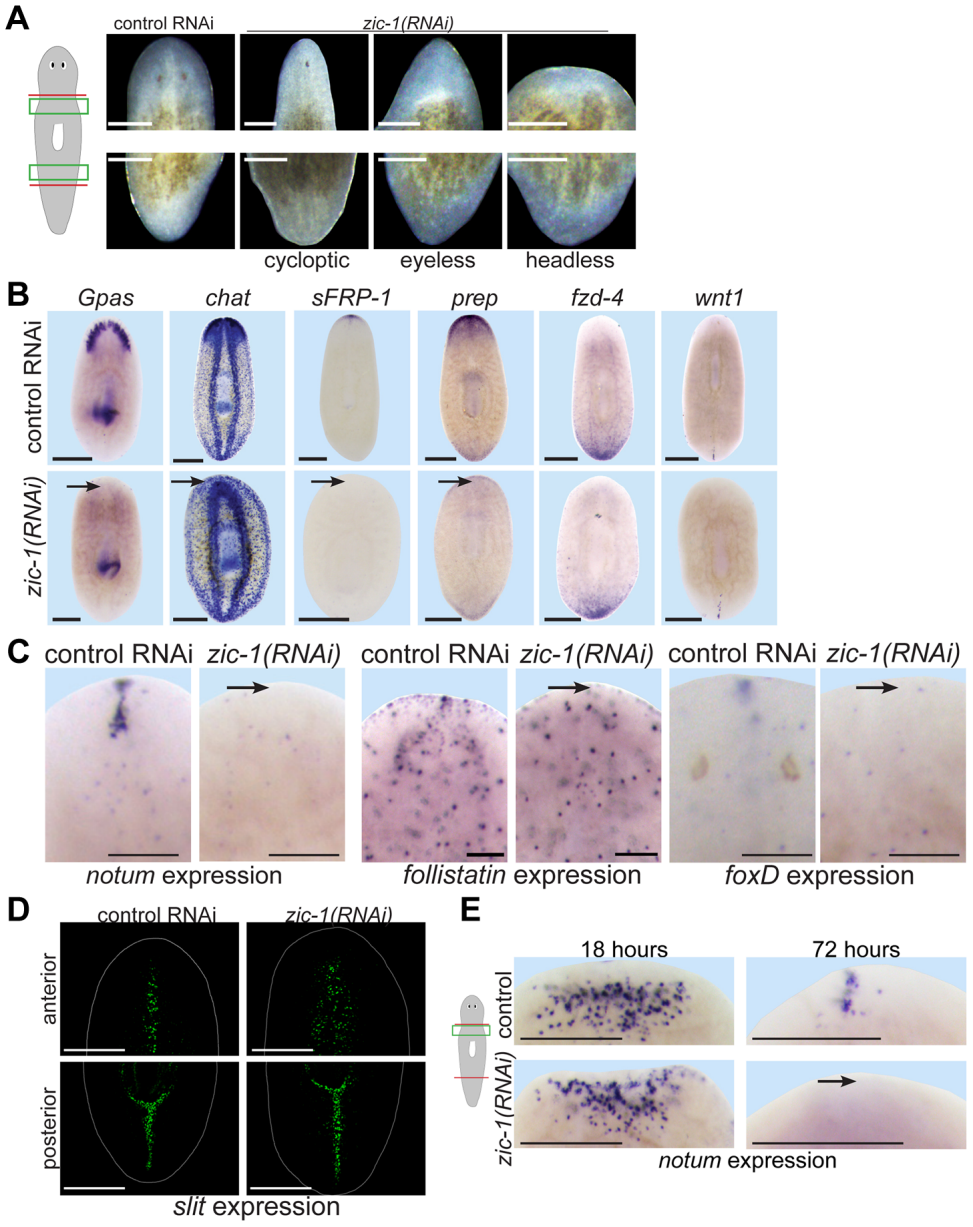
*zic-1* is required for anterior pole formation and head regeneration. (A) Animals 8 days after amputation of heads and tails after treatment with control dsRNA or *zic-1* dsRNA, cartoon shows enlarged regions of the anterior and posterior blastemas. Inhibition of *zic-1* function caused anterior regeneration defects such as cyclopia (24%, n = 74 animals scored), lack of eyes (12%, n = 74 animals scored), and head regeneration failure (63%, n = 90 animals scored). (B–D) in situ hybridizations of animals fixed 8 days after amputation. (B) The anterior region of headless *zic-1(RNAi)* regenerating animals lacked a brain (*gpas*, 2/2 animals probed; *chat*, 4/4 animals probed) and expression of head tip marker (*sFRP-1*, 11/11 animals probed) and head region marker (*prep*, 8/8 animals probed) and did not express posterior markers (*fzd-4*, 7/7 animals probed; *wnt1*, 7/7 animals probed). (C) *zic-1(RNAi)* animals failed to express *notum* (6/6 animals probed), *follistatin* (4/5 animals probed), and *foxD* (14/14 animals probed) at the anterior pole after 8 days of regeneration. (D) *zic-1(RNAi)* animals had a laterally expanded midline in their anterior but not their posterior, as assayed by *slit* expression (4/4 animals). (E) *zic-1* is required for anterior pole formation by 72 hours and not for early wound-induced *notum* expression at 18 hours of regeneration. In situ hybridizations to detect *notum* expression after treatment with control or *zic-1* dsRNA, amputation of heads and tails, and fixation at 18 or 72 hours. *zic-1* inhibition prevented expression of *notum* at the anterior pole by 72 hours (8/8 animals) but had no effect on its expression at 18 hours (122±53 *notum+* cells in control and 108±35 *notum+* cells in *zic-1(RNAi)* animals, 8 animals probed, p = 0.59 t-test). Cartoon shows surgery and enlarged regions. Bars, 100 microns (C), 250 microns (A), 300 microns (B, D, E).

We next used RNAi to investigate functions for *zic-1* in regeneration. Animals treated with *zic-1* double-stranded RNA (dsRNA) were amputated to remove heads and tails and scored for phenotypes 8 days after surgeries. Inhibition of *zic-1* caused overt defects in head but not tail regeneration, including cyclopia (25% of 74 animals assayed), absence of eyes (12% of 74 animals assayed) and head regeneration failure (63% of 90 animals assayed) (Fig. 4A). Two dsRNAs targeting non-overlapping regions of the *zic-1* gene individually caused identical defects in anterior regeneration, suggesting these effects are likely due to *zic-1* inhibition and not an off-target effect of RNAi (5′ dsRNA: 7/14 animals were headless; 3′ dsRNA: 3/6 animals were headless). Similar anterior regeneration defects have been observed due to inhibition of *notum* [14], *foxD* [41], *follistatin* [24], [25], *patched* [15], *prep* [56], *pbx* [37], [38], and *H+,K+-ATPase* [30]. We used in situ hybridizations to analyze the tissue content and regional expression status of headless *zic-1(RNAi)* animals. Headless *zic-1(RNAi)* animals lacked a brain (*gpas, chat*) and also lacked expression of markers of the head tip (*sFRP-1*), head region (*prep*), and anterior pole (*notum*, *follistatin* and *foxD*) (Fig. 4B–C). Headless (Fig. 4B) and eyeless (Fig. S4A) *zic-1(RNAi)* animals had normal expression of posterior markers *fzd-4* and *wnt1*, suggesting that posterior regeneration occurred normally and that such animals did not undergo a polarity transformation to form a tail at the anterior-facing wound sites. To further test whether *zic-1* has functions in midline formation, we analyzed expression of *slit*, a marker of the midline [57]. *zic-1(RNAi)* animals had laterally expanded anterior put not posterior expression of *slit* (Fig. 4D). *zic-1(RNAi)* animals maintained broad expression of *pbx* (Fig. S4B), indicating *zic-1* likely does not function in head regeneration by regulating *pbx* expression. Together, these results indicate *zic-1* participates in head formation rather than pole identity determination.

*zic-1* inhibition resembled some defects due to inhibition of *notum*, so we determined the stage(s) of *notum* expression affected by *zic-1* RNAi. *zic-1* inhibition prevented anterior pole expression of *notum* by 72 hours, but not its early wound-induced expression (Fig. 4E), similar to the impact of irradiation on *notum* expression (Fig. 1A). Together, these experiments indicate *zic-1* is required for head regeneration, midline patterning, and anterior identity.

*zic-1* inhibition produced multiple regeneration phenotypes, so we sought to understand their relationships by performing RNAi attenuation experiments and additional histological analysis. Dilution of *zic-1* dsRNA with an equal amount of control dsRNA increased the penetrance of cyclopia and decreased the penetrance of headlessness (Fig. S4C). In addition, cycloptic *zic-1(RNAi)* animals had a low level of *notum* and *sFRP-1* expression at the anterior pole (Fig. S4D). We conclude that cyclopia is likely a hypomorphic phenotype due to *zic-1* RNAi and we did not investigate it further.

An additional Zic-family gene in the *S. mediterranea* genome, formerly referred to as *zicB*, we renamed *Smed-zic-2* (here after referred to as *zic-2*) in keeping with the *S. mediterranea* guidelines for gene names [53]. z*ic-2* was expressed in the anterior of uninjured animals (Fig. S5A), and we speculated that it could have redundant functions with *zic-1*. Double fluorescence in situ hybridizations revealed that *zic-2* was expressed in neoblasts at 24-hours and in cells of the anterior region at 72-hours in an irradiation sensitive manner (Fig. S5B). Inhibition of *zic-2* by RNAi resulted in weakly penetrant cyclopia after amputation and regeneration (Fig. S5C). However, simultaneous inhibition of *zic-1* and *zic-2* increased the frequency of headless animals versus inhibition of *zic-1* or *zic-2* alone (p-value<0.001, Fisher's exact test). *zic-2* RNAi reduced expression of injury-induced *zic-1* (p = 0.07), as measured by manual cell scoring and realtime PCR (Fig. S5D–E), providing a possible explanation of these effects given that dsRNA treatments in general likely reduce rather than eliminate gene function. By contrast, *zic-1* RNAi caused a reduction in *zic-2* expression (p = 0.07) (Fig. S5E). Taken together, these results suggest *zic-1* and *zic-2* function together in head regeneration. Because head regeneration strongly required *zic-1*, we focused subsequent analysis on that gene.

### *zic-1* has injury-independent functions in anterior pole maintenance

Given the prominent injury-induced behavior of *zic-1* expression, we hypothesized that it may have functions specific to regeneration as opposed to those tissue maintenance in the absence of injury, similar to *follistatin* [25], *runt-1* [9], and *foxD* [41]. To test whether *zic-1* functions only in regeneration, we fed animals bacteria expressing dsRNA for 10 weeks. No overt abnormalities became apparent in these animals (Fig. S6A), and *sFRP-1* expression was normal (Fig. S6B). However, prolonged *zic-1* RNAi without injury caused a reduction in the number of *notum*+ cells near the anterior pole (11±1 *notum+* cells in control animals versus 5.8±0.5 cells in *zic-1(RNAi)* animals, p<0.005 by t-test) (Fig. S6B). This defect was specific to expression of *notum* at the anterior pole, as *notum* expression at the brain commissure was normal (10.7±2.3 *notum+* cells in control animals versus 13±1.6 cells in *zic-1(RNAi)* animals, p>0.05 by t-test). By contrast, amputation of animals undergoing RNAi in parallel treatments for only three weeks resulted in head regeneration failure (40%, n = 20 anterior-facing wounds). These results point to the importance of injury-induced *zic-1* expression for regeneration and indicate injury-independent functions in anterior pole maintenance.

### *zic-1* allows sustained Wnt signaling inhibition needed for head regeneration

**Figure 5:**
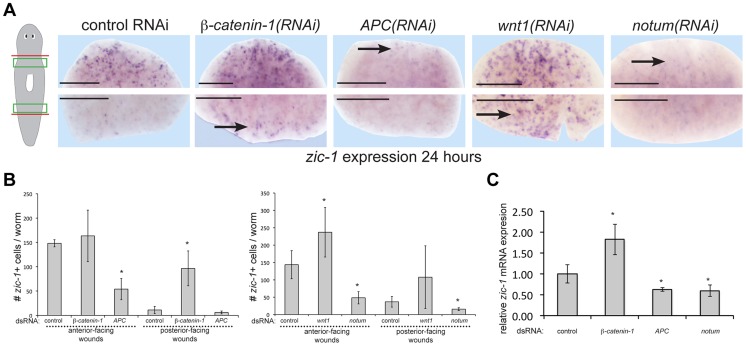
Polarized expression of *zic-1* early after injury is controlled by *notum*'s inhibition of canonical Wnt signaling. (A) *beta-catenin-1* or *APC* were inhibited by feeding animals bacteria expressing dsRNA four times over two weeks and *notum* or *wnt1* were inhibited by injection of dsRNA. Animals were fixed 24 hours after amputation of heads and tails and stained with a *zic-1* riboprobe. Cartoon shows surgery and enlarged region. *beta-catenin-1* inhibition caused ectopic expression of *zic-1* at posterior-facing wound sites, whereas *APC* inhibition caused a reduction of *zic-1* expression at anterior-facing wound sites. Similarly, *wnt1* inhibition increased *zic-1* expression at anterior- and posterior-facing wound sites, whereas *notum* inhibition decreased its expression at both wound sites. (B) Quantitation of number of cells expressing *zic-1* under each treatment. Error bars standard deviations, asterisks p-value<0.05 by t-test. (C) qPCR assays were performed on total RNA isolated with 4 biological replicates of three worms each to measure *zic-1* expression relative to *gapdh* after the indicated RNAi treatments. Error bars standard deviations, asterisks p-value<0.05 by t-test. Scale bars, 250 microns.

We next examined determinants of *zic-1* expression and function. Expression of some Wnt signaling ligands and secreted inhibitors occurs independently of neoblasts and also with A/P polarization at injury sites [13], [32]. Therefore, we tested canonical Wnt signaling as a candidate pathway controlling early *zic-1* activation (Fig. 5A–B). Inhibition of *beta-catenin-1* by RNAi resulted in ectopic *zic-1* expression at posterior-facing wounds in trunk fragments by 24 hours after injury. Conversely, inhibition of *APC*, an intracellular inhibitor of *beta-catenin-1*, reduced *zic-1* expression at anterior-facing wound sites in trunk fragments. Because *zic-1* expression behavior depends on A/P axis position (Fig. 2A), we further investigated the impact of *beta-catenin-1* or *APC* inhibition in anterior-facing wound sites from anterior- and posterior-facing injury amputation sites at different A/P axial positions (see cartoons to show surgeries in Fig. 5A–B, S7A–B). *APC* inhibition decreased *zic-1* expression at anterior-facing injury sites in both the anterior and posterior of the body, and also in posterior-facing injury sites from the anterior of the body (Fig. 5A–B, S7A–B). By contrast, *beta-catenin-1* inhibition increased *zic-1* expression at anterior- and posterior-facing amputation sites located in posterior regions of the animal but not in the anterior of the animal (Fig. 5A–B, S7A–B). Taken together, these results indicate that perturbation of *beta-catenin* or *APC* function has opposite effects on *zic-1* expression that are independent of polarity transformations caused by these treatments. Animal-wide gradients of *beta-catenin* activity have been proposed based on phenotypic evidence and expression domains of Wnt ligands [58]. We speculate that anterior regions may normally have sufficiently low *beta-catenin-1* activity that *beta-catenin-1* RNAi does not further increase *zic-1* expression after injury. Furthermore, in uninjured animals, *beta-catenin-1* or *APC* RNAi did not simply result in constitutive activation or inhibition of *zic-1* in the regions assessed in the regeneration assay (4/4 animals scored, Fig. S7C), indicating these effects were specific to an injury context.

*wnt1* and *notum* are expressed prior to *zic-1* activation in response to injury, so we tested their requirements for *zic-1* expression. *wnt1* RNAi resulted in ectopic *zic-1* expression at posterior-facing amputation sites, and *notum* RNAi reduced expression at anterior-facing amputation sites. We confirmed the effects of Wnt signaling perturbation on *zic-1* expression using manual cell counting (Fig. 5B) and qPCR (Fig. 5C). Therefore, taken together with previous observations, these results indicate that Wnt signaling inhibition by early injury-induced *notum* is necessary and sufficient to activate early *zic-1* expression by 24 hours.

We examined additional genes involved in head and tail regeneration for their requirements for 24-hour *zic-1* expression. Inhibition of *hedgehog* and *patched* had no apparent effect on *zic-1* expression as measured by qPCR and did not alter numbers of *zic-1+* cells as measured by manual counting (Fig. S8A). *hedgehog* and *patched* oppositely regulate *wnt-1* expression, suggesting that transcriptional activation of *zic-1* could have additional inputs other than Wnt signaling. However, *patched(RNAi)* animals of the same cohort regenerated with anterior defects, including anterior tail formation (3/7 animals) and headlessness (2/7), suggesting that Wnt signaling inhibition rather than axis polarization or regionalization is a driver of *zic-1* expression due to injury. Additionally, *pbx* inhibition reduced but did not eliminate *zic-1* expression 24 hours after amputation, as measured by reduced numbers of *zic-1+* cells and reduced *zic-1* mRNA levels (Fig. S8B–C). *follistatin* inhibition reduced *zic-1+* cell numbers at 24 hours after amputation and reduced *zic-1* mRNA levels, although not significantly as determined by a t-test, consistent with *follistatin* participating in early signaling due to any injury that removes tissue [25]. *pitx* inhibition reduced the numbers of *zic-1*+ levels, although not significantly as determined by a t-test, and reduced *zic-1* mRNA expression. *foxD* inhibition did not visibly alter *zic-1* expression or numbers of *zic-1*+ cells at 24 hours, though reduced mRNA expression of *zic-1* but not significantly (as determined by t-test). Additionally, *prep* RNAi did not alter numbers of *zic-1+* cells nor mRNA levels due to injury expression. Finally, *pitx* RNAi reduced the numbers of *zic-1+* cells and reduced *zic-1* mRNA levels. We conclude that *pbx*, *follistatin* are likely required for maximal levels of *zic-1* expression, and that *zic-1+* cells are still abundant after inhibition of *hedgehog*, *patched*, *foxD*, *pitx* and *prep*.

**Figure 6:**
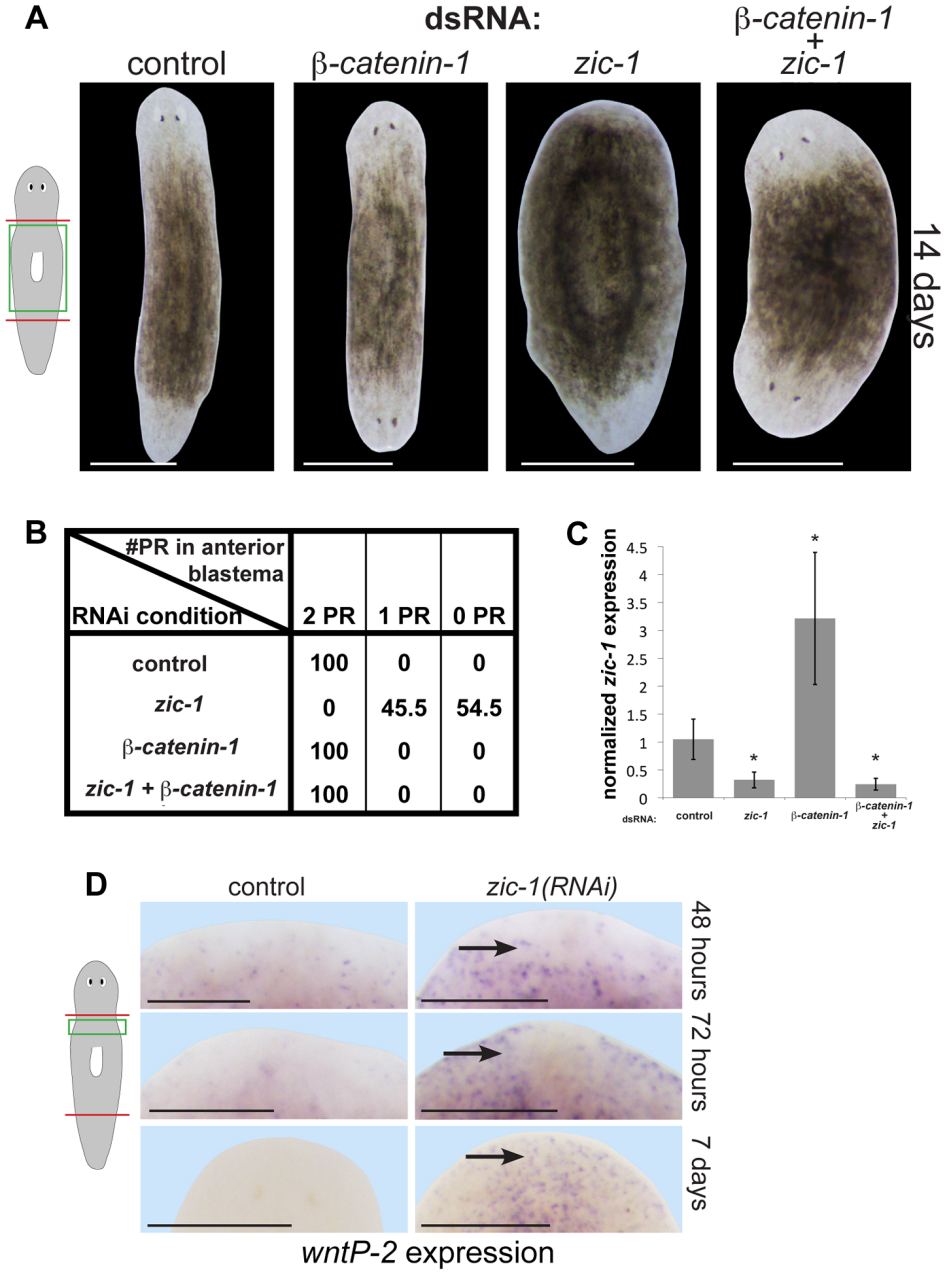
*zic-1* inhibits Wnt signaling to promote head outgrowth. (A) Single and double-RNAi as indicated to examine interactions between *zic-1* and *beta-catenin-1*. Total concentrations of dsRNA were normalized by control dsRNA so that the absolute amount of each utilized dsRNA was equivalent across treatments. *zic-1(RNAi);beta-catenin-1(RNAi)* animals all regenerated heads after decapitation suggesting that *beta-catenin-1* inhibition is required for the *zic-1*'s head-promoting function. (B) Scoring percent of anterior regeneration blastema with 0, 1, or 2 photoreceptors (PR) (n>9 animals). (C) qPCR analysis of *zic-1* expression versus *gapdh* in animals treated with dsRNA as indicated. *zic-1* dsRNA reduced *zic-1* mRNA levels (p<0.05, t-test), *beta-catenin-1* dsRNA increased *zic-1* mRNA (p<0.05, t-test), and simultaneous treatment with *zic-1* and *beta-catenin-1* dsRNA decreased *zic-1* mRNA (p<0.01, t-test). (D) *wntP-2*, a gene normally expressed in the posterior by *beta-catenin-1* activity, was ectopically expressed in *zic-1(RNAi)* animals by 48–72 hours after decapitation, and maintained 7 days after amputation. Taken together, *zic-1* inhibits *beta-catenin-1* to promote head formation. Scale bars, 300 microns (D) or 500 microns (A).

Given the central function of Wnt signaling in head regeneration [10], [11], [59], we next tested candidate functional interactions with *zic-1*. Whereas *zic-1* promotes head regeneration (Fig. 4A), *beta-catenin-1* suppresses it [10], [11], [59], so we examined the outcome of simultaneous inhibition of both genes. *zic-1(RNAi);beta-catenin-1(RNAi)* animals all regenerated heads at anterior-facing wounds and posterior-facing wounds, identical to *beta-catenin-1(RNAi)* animals (Fig. 6A–B). qPCR performed on RNA from animals of the same cohort harvested 24 hours after amputation in biological triplicate verified *zic-1* transcript reduction by *zic-1* dsRNA treatments (Fig. 6C). These experiments indicate the suppressive effect of *beta-catenin-1* dsRNA was not simply due to reduction in *zic-1* RNAi efficiency. Additionally, we used in situ hybridizations to measure *notum* expression in animals from this experiment fixed at 72 hours and 14 days of regeneration (Fig. S9A). *beta-catenin-1* inhibition reduced anterior pole expression of *notum* at 72 hours, consistent with *notum's* described property as a feedback inhibitor of Wnt signaling. Such animals regenerate an anterior head with reduced numbers of *notum+* cells at the anterior pole by 14 days. *zic-1* inhibition eliminated *notum* expression at the pole by 72-hours. Simultaneous inhibition of *beta-catenin-1* and *zic-1* resulted in head regeneration but in the absence of *notum* expression at 72 hours.

These results indicate that experimental inhibition of *beta-catenin-1* can promote head regeneration in the absence of *zic-1* and *notum*, likely by fulfilling the normal function of anterior pole-expressed *notum* as a Wnt inhibitor. In support of the first model, *wntP-2/wnt11-5*, a gene whose expression is activated by Wnt signaling in the posterior [13], is inappropriately expressed in the anterior of *zic-1(RNAi)* animals by 48/72 hours and even 7 days after decapitation (Fig. 6D), suggesting *zic-1* inhibits *beta-catenin-1* signaling. *notum* is required for anterior polarity and head regeneration whereas *zic-1* is required only for head regeneration, and this distinction may account for the difference in extent of ectopic anterior *wntP-2* expression observed after inhibition of the two genes [14]. We additionally found that *zic-1* RNAi had no effect on *beta-catenin-1* mRNA levels (Fig. S9B). *beta-catenin-1* shares a similar epistatic relationship to *zic-1* and *notum*, and *zic-1* is required for *notum* expression, so we propose that injury-induced *zic-1* promotes *notum* expression at the anterior pole which in turn represses Wnt signaling to allow head formation. We suggest that early, *zic-1*-independent, wound-induced *notum* expression is required for anterior polarity, whereas later *zic-1*-dependent *notum* expression at the pole orchestrates head patterning and outgrowth.

### *zic-1* controls a neoblast specification program that forms the anterior pole as an early step in head regeneration

*zic-1*'s expression in neoblasts suggested it might function to control neoblast behaviors relevant for pole formation and/or specification of anterior cell types. Notably, however, tail regeneration and *wnt1* expression at the posterior pole, both neoblast-dependent processes, occurred normally in *zic-1(RNAi)* animals (Fig. 4A–B), indicating *zic-1* is not required for generic neoblast function. Additionally, *zic-1(RNAi)* animals had abundant *smedwi-1* expression in both the anterior and posterior (Fig. S10A), indicating this gene is not required for regional maintenance of neoblasts in the anterior.

**Figure 7:**
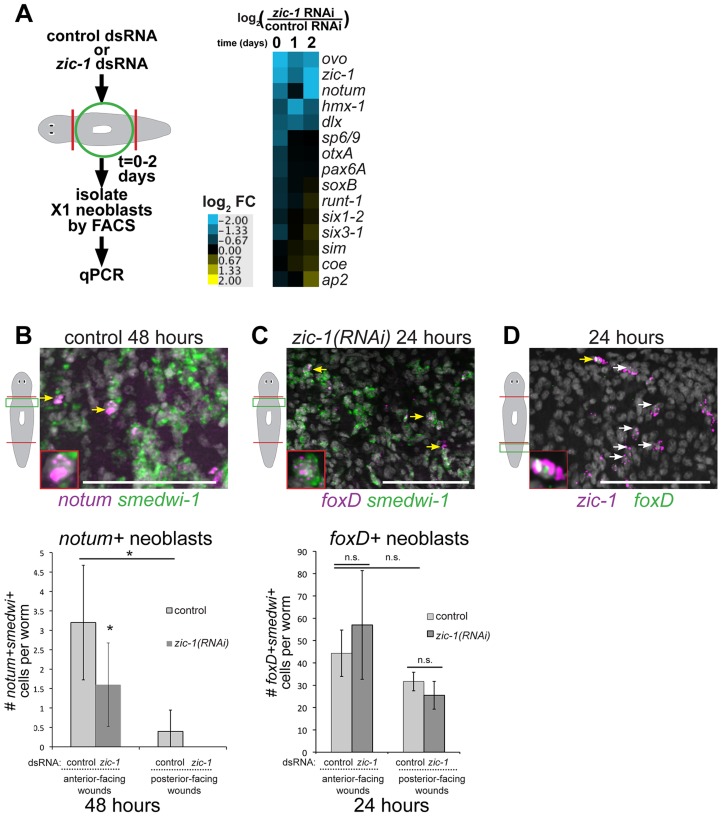
*zic-1* controls utilization of *foxD+smedwi-1+* progenitors for *notum* expression and anterior pole formation. (A) Measurement of expression changes in X1 neoblasts due to treatment with *zic-1* versus control dsRNA. Trunk fragments were macerated 5 minutes (0 days), 24 hours (1 day) and 48 hours (2 days) after head and tail amputation, and X1 cells were isolated from 3 independent biological replicates of 10 worms each. qPCR examined putative expression changes in a cohort of 15 transcription factors and signaling molecules that mark neoblast subpopulations induced by regeneration. Cartoon depicts experimental design. Heat map shows log_2_ fold-changes in gene expression for the indicated genes in *zic-1(RNAi)* X1 neoblasts versus controls averaged from three biological replicates for each condition. Blue color indicates downregulation and yellow indicates upregulation, due to *zic-1* RNAi. (B–D) Double FISH to detect expression of *zic-1*, *foxD*, *smedwi-1* and/or *notum* with nuclei counterstained with Hoechst (gray). (B) In situ hybridization probing to detect *notum* (magenta) and *smedwi-1* (green) to detect *notum+smedwi-1+* cells. (B, top) Representative image of *notum+smedwi-1*+ cells, (B, bottom) quantification of *notum+smedwi-1+* cells. *zic-1(RNAi)* reduced numbers of *notum+* neoblasts by 48 hours and *smedwi-1+* cells were more numerous in anterior than posterior. (C) *foxD* (magenta) expression in *smedwi-1+* (green) neoblasts 24 hours after injury was not eliminated due to *zic-1* RNAi. Bottom, quantification of *foxD+smedwi-1+* cells from control and *zic-1(RNAi)* animals fixed 24 hours after head and tail amputation and probed by double FISH. *foxD+smedwi-1+* cells were produced in equal numbers at anterior- versus posterior-facing wound sites and were not reduced by *zic-1* inhibition (control animals, 29±1 *foxD+smedwi-1+* cells in anterior- versus 32±4 in posterior-facing wounds; *zic-1* RNAi animals, 19±5 *foxD+smedwi-1+* cells in anterior- versus 26±6 in posterior-facing wounds, n = 3 worms each). (D) *foxD* (green) is expressed in a subpopulation of *zic-1+* cells (magenta, 3.4±1.8% of 420 *zic-1*+ cells were *foxD*+, n = 5 worms) by 24 hours. Yellow arrow indicates double positive cell (also inset), and white arrows indicate *zic-1+* cells. Cartoons show surgeries and zoomed areas. Error bars standard deviations, asterisks p-value<0.05 by t-test. Bars, 75 microns.

We next tested for possible involvement of *zic-1* in neoblast specification programs. We purified total RNA from X1 neoblasts isolated by FACS from animals treated with *zic-1* or control dsRNA in a time series and analyzed expression of 15 transcription factors and signaling molecules described to be activated in neoblast subpopulations due to regeneration (heat map shown in Fig. 7A with fold-changes and p-values shown in Table S2). *zic-1* RNAi caused a significant (t-test p-value<0.05) reduction greater than 2-fold in expression of *ovo* (0, 24 and 48 hours), *zic-1* (0, 24, 48 hours), *notum* (48 hours) and *distalless* (*dlx*) (24 hours). *zic-1* RNAi reduced *hmx-1* expression although not significantly (p = 0.11 at 24 hours), and significantly elevated expression of *ap2* by 1.7-fold (48 hours). Several genes did not have significant fold-changes greater than 1.7-fold (p>0.05, t-test): *hmx-1*, *sp6/9*, *OtxA*, *pax6A*, *soxB*, *runt-1*, *six1-2*, *six3-1*, *sim*, and *coe*. We confirmed the observations of this expression analysis for selected genes using in situ hybridizations. *zic-1* inhibition reduced *ovo* expression by 48 hours (Fig. S10B) and had minimal effects on *runt-1* by 24 hours (Fig. S10C). Because *runt-1* is also activated in neoblasts early (by 3 hours) in response to injury, we examined candidate reciprocal interactions with *zic-1*. However, *runt-1* RNAi did not alter expression of wound-induced *zic-1* (Fig. S10D). Therefore, *zic-1* and *runt-1* likely do not regulate each other transcriptionally. Finally, *ap2* was induced by injury and expressed highly in the anterior of *zic-1* RNAi animals (Fig. S10E), indicating that *zic-1* RNAi does not simply result in a generic failure of neoblast functions in the anterior. These results indicate *zic-1* is needed for expression of *notum*, *ovo* and *distalless* within the neoblast population and highlight the specificity of these functions.

To identify neoblast responses regulated by *zic-1* and specific for anterior pole formation, we turned our attention to *notum* and *foxD*, which are both needed for head formation and expressed in subpopulations of neoblasts during regeneration. *notum* expression was reduced by 48 hours in the analysis of X1 cells from *zic-1(RNAi)* animals (Fig. 7A), suggesting that *zic-1* may control anterior pole formation by regulating specification of *notum* in neoblasts. To confirm this prediction, we analyzed *zic-1(RNAi)* versus control animals at 48 hours of regeneration using double FISH and counting of *notum+*/*smedwi-1+* neoblasts. *zic-1(RNAi)* animals had reduced numbers of *notum+*/*smedwi-1+* cells, indicating *zic-1* is required for specification of *notum+smedwi-1+* cells in addition to expression of *notum* in *smedwi-1−* cells of the anterior region (Fig. 7B, Fig. 4E). We next examined the relationship between *zic-1* and *foxD*. *foxD* expression is irradiation sensitive by 24 hours and co-expressed in a neoblast subpopulation believed to participate in pole formation [41]. *zic-1* inhibition did not alter the abundance of *foxD+*/*smedwi-1+* neoblasts at anterior-facing wound sites by 24 hours (Fig. 7C). In addition, *foxD* is expressed in a subpopulation of *zic-1+* cells at 24 hours, with 66% of *foxD+* cells co-expressing *zic-1* (14/21 cells, n = 5 worms), and 3% of *zic-1+* cells co-expressing *foxD* (14/420 cells, n = 5) (Fig. 7D). We did not observe a general requirement of *foxD* for 24-hour *zic-1* expression (Fig. S8B–C), but we cannot rule out the possibility that *foxD* is required for *zic-1* expression in this small minority of *zic-1+* cells that co-express *foxD*. Taken together, these results suggest *zic-1* is not likely needed for specification of *foxD+* neoblasts but rather for their utilization or further specification to form *notum+* neoblasts that regenerate the anterior pole.

We further noted differences in the abundance of these progenitor cells at anterior- and posterior-facing wounds important for considering their relationships. *foxD+* neoblasts were present at both anterior- and posterior-facing wounds in similar numbers (p-value>0.05, t-test) (Fig. 7C). By contrast, *zic-1* expression in neoblasts was polarized for anterior-facing injury sites at 24 hours (Fig. 1C–D), and *notum+* neoblasts were enriched near anterior-facing wounds versus posterior-facing wound sites (Fig. 7B). These results suggest that *zic-1* may act as a polarizing cue to promote *foxD* progenitors to undergo anterior pole cell specification only at anterior-facing injury sites.

## Discussion

**Figure 8:**
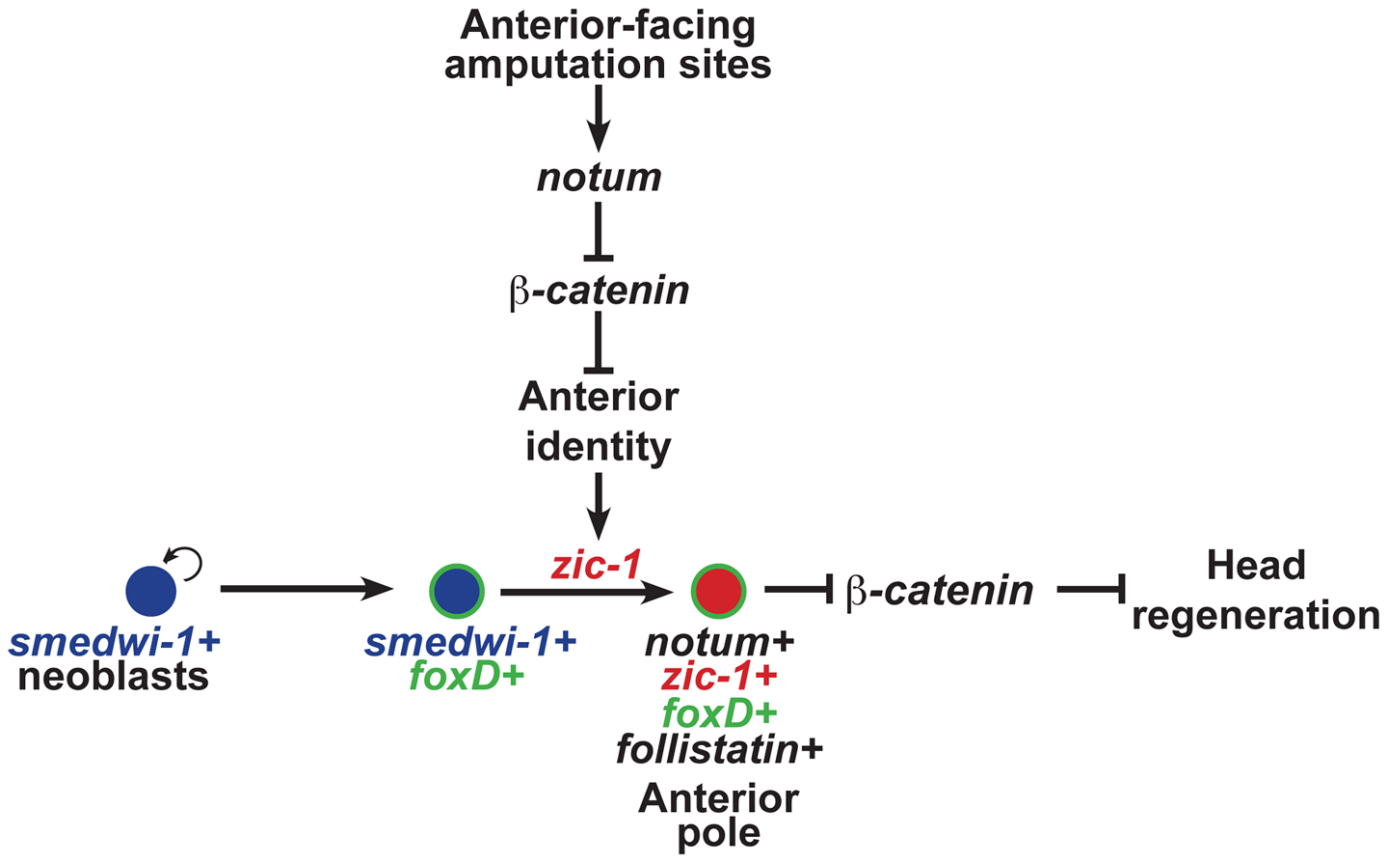
*zic-1* function couples injury signaling and polarized Wnt signaling cues for activation of stem cells to produce the anterior pole in early head regeneration. Anterior pole formation is neoblast-dependent, specific to anterior-facing amputations and midline cues, and dependent on *foxD* and *zic-1*. Prior to 24 hours, injury-induced expression of *notum* and *wnt1* (not depicted) in *collagen*+ body wall muscle cells engages a decision process that determines anterior versus posterior identity at an amputation site. At anterior-facing amputation sites, this results in a low canonical Wnt signaling environment which promotes anterior identity and, directly or indirectly, expression of *zic-1* in a population of *smedwi-1+* neoblasts, some of which are *foxD+*. By 48 hours, *zic-1* promotes specification of *notum+smedwi-1+* progenitors that form an anterior pole signaling center that expresses *zic-1*, *foxD*, *follistatin* and *notum*. Signaling from the anterior pole, likely including *notum* activity, suppresses Wnt signaling to promote head outgrowth and patterning. Experimental inhibition of *beta-catenin-1* can fulfill the signal inhibitory function of *notum* necessary for head formation in the absence of *zic-1* or the anterior pole. *zic-1* expression preferential to anterior-facing amputation sites ensures anterior-specific utilization of *foxD+smedwi-1+* progenitors that are formed at both anterior and posterior injury sites.

Tissue organizers orchestrate growth and patterning in embryonic development, but it is not clear whether or how such signaling centers could contribute to coordinated growth in adulthood. In adult regeneration, stem cells or other progenitor cell types provide a supply of new differentiating cells necessary to replace missing structures, but such cells likely require instructive information prompted by injury in order to construct complex tissues. Here we describe the function of a Zic family transcription factor that couples injury signaling and polarized Wnt signaling cues [10], [11], [13], [14], [32], [59] for activation of stem cells to produce an anterior signaling center needed for head regeneration (Fig. 8).

The functional and expression data support a model for head regeneration in which an initial anterior polarity decision, controlled by injury-induced *notum*, activates *zic-1* expression in neoblasts, which enables head regeneration by production of *notum*+ cells of the anterior pole. Before 24 hours at anterior-facing amputation sites, *notum* is activated in cells of the body-wide musculature where it inhibits injury-induced *wnt1* to create a low Wnt signaling environment, anterior identity, and subsequently *zic-1* expression in neoblasts. By contrast, posterior-facing injury sites do not abundantly express *notum*, allowing injury-induced *wnt1* signaling to predominate and enforce high levels of *beta-catenin-1* signaling that ultimately suppress *zic-1* expression. Anterior- or posterior-facing injury sites have different *zic-1* expression responses according to their position along the A-P axis. We suggest that the initial expression of *zic-1* at the posterior-facing wound sites of head fragments reflects a low Wnt signaling environment present at the time of injury, but in such fragments *zic-1* expression is ultimately suppressed as the region takes on posterior polarity and identity through *wnt1* signaling. *foxD* is expressed in neoblast subpopulations at both anterior- and posterior-facing amputation sites. *zic-1* activity at anterior-facing wound sites triggers further specification or utilization of *foxD*-expressing neoblasts for expression of *notum* and subsequent anterior pole formation by 48 hours. *zic-1* is neoblast-expressed early and co-expresses with *foxD* in pole progenitors at the anterior pole, so the most likely scenario is that it acts autonomously to control this specification process, but we cannot rule out possible indirect functions for *zic-1* in head regeneration or patterning. The regenerating anterior pole produces NOTUM protein, which likely sustains a low Wnt signaling environment, possibly through positive feedback by specification of additional *zic-1+* neoblasts that continue to contribute new cells to the anterior pole to promote head regeneration.

Injuries can alter tissue content in numerous ways, so regenerating animals must have mechanisms that robustly allow production of appropriate missing structures. It is difficult to envision a complex contingency system that continuously probes each tissue type for its presence or absence to address this need. Alternatively, a relatively simple system that utilizes information about the position, orientation, and extent of a wound site could be sufficient for achieving this goal. *zic-1* expression activation after wounding depends both on the injury's A/P axial location (Fig. 2F) as well as its A/P orientation (Fig. 2B), suggesting the existence of such systems. It is also possible that *zic-1* expression is activated at other sites or tissues requiring maintenance of low Wnt signaling, as we observed expression of *zic-1* in some non-anterior regions during regeneration, similar to *notum* [14]. However, sustained and abundant *zic-1* expression is enriched at anterior-facing amputation sites (Fig. 2B). Wound-induced signaling through 6–24 hour expression of *wnt1* and *notum* may have a particular importance in regeneration scenarios that require significant alteration of A/P tissue identity. Examining the relationship between pre-existing regional cues and wound-activated signaling will be important for understanding how regenerative outgrowth is achieved.

Planarian transcription factors have been identified that function in either neoblast subpopulations for specification of specific differentiated cell types or function broadly in the process of axis formation. Several lineage-specific transcription factors are expressed within subpopulations of neoblasts for forming cells of the eye, protonephridia, intestine, brain [3], [51], [52], [60]–[62] and additional post-mitotic progeny [55], [63]–[65]. For example, *runt-1* is expressed within neoblasts [9] and is required for eye formation [66]. By contrast, transcriptional regulators have also been implicated in axis formation. Tail regeneration and expression of *wnt1* in the tail requires *islet1*, a transcription factor expressed in the posterior [67], as well as *pitx*, expressed at the anterior and posterior poles [39], [40]. The TALE homeobox gene *prep* is needed for head formation [56], and *pbx*, another TALE transcription factor, is needed for both head and tail formation [37], [38]. Our results identify *zic-1* as an injury-induced neoblast-expressed gene with possible related functions in both cell specification and axis formation.

The participation of vertebrate Zic proteins in multiple developmental processes and the existence of several Zic family members have complicated efforts to identify their common use in only a single signaling pathway, and interactions with Hedgehog, Wnt, Activin, and retinoic acid signaling have been reported [50], [68]. Early wound-induced *wnt1* expression that occurs by 18 hours after injury is dependent on *hedgehog* signaling, and still occurs in irradiated animals [13], [32] that lack *zic-1* expression (Fig. 2E). Therefore, neoblast-expressed *zic-1* is unlikely to be a general regulator of Hedgehog signaling. Our results indicate repression of Wnt signaling, possibly through expression of signaling inhibitors, could be an ancient and conserved function for Zic proteins. Additionally, Activin signaling repression by *follistatin* is needed for both head and tail regeneration in planarians [25], suggesting that *zic-1* is not a general regulator of that pathway in planarians. Vertebrate Zic2 and Zic3 regulate organizer function by inhibition of Wnt signaling [42], [69]. Additionally, mutations in human *zic2* lead to holoprosencephaly [70], a midline defect of the anterior due to perturbed organizer function [43]. Our studies of planarian *zic-1* suggest Zic proteins may have had ancient functions and conserved functions in Wnt signaling inhibition involved in injury responses or control of tissue organizing regions.

In principle, the positional information necessary for regeneration could either be dependent or independent of stem cell function. Planarians depleted of neoblasts maintain the ability to undergo some generic injury-induced signaling and also signaling polarized along the body axes [9], [13], [32]. By contrast, our analysis of *zic-1* suggests that a critical step in blastema formation is the production of new differentiated cells to perform signaling functions that subsequently direct the activity of additional cells for tissue outgrowth. Programs that use stem cells or specialized progenitors to produce signaling centers after injury may be utilized in general for normal regenerative growth. The use of stem cells to construct tissue organizers post-embryonically could facilitate the synthesis or repair of non-regenerative organs.

## Materials and Methods

### Animals and irradiation treatments

Animals of the asexual strain of the planarian *Schmidtea mediterranea* were maintained in planarian water (1× Montjuic salts) at ∼19°C as previously described [14]. Planarians were fed a liver paste and starved for at least seven days before experiments. Where indicated, animals were gamma-irradiated with a lethal dose of 6,000 Rads using a Cesium source irradiator 48 hours before surgeries (Fig. 1A, S1, S2A and S5B) or the day of amputation (Fig. 2E).

### FACS sorting, RNA extraction and microarray analysis

For gene expression profiling by microarray (Fig. 1C) and realtime PCR (Fig. 1D), neoblasts were isolated from tissue near wound site by FACS in a time series (0, 24, 48 and 72 hours post amputation). For each biological replicate, tissue from anterior- or posterior-facing injury sites from 8 animals was collected, macerated, labeled with Hoechst 33342 and propidium iodide and sorted as previously described [64]. For each biological replicate, 40,000–100,000 cells were collected into Trizol-LS, and RNA was extracted using RNeasy Plus Mini Kit (Qiagen, 74134). Libraries were prepared using a WTA2 amplification kit (Sigma), and arrays were hybridized and scanned according to the manufacturer's instructions at the Washington University Genome Technology Access Center. Custom oligonucleotide arrays from Agilent had 140,000 probes intended to represent the majority of *S. mediterranea* genes identified through previous EST sequencing, RNA-seq and transcriptome assembly, and gene predictions [53], [64], [66], [71], [72]. The limma Bioconductor software package (Linear Models for Microarray Data) implemented in R was used for analysis of single-channel microarray data [73]. Briefly, array intensities were background subtracted (method = “normexp”, offset = 16), quantile-normalized between arrays, and differential gene expression was evaluated using eBayes which produces for each gene a t-statistic of the ratio of the log2-fold change to the standard error moderated across all genes in the experimental series to obtain a p-value that was used to compute a false-discovery rate by correcting for multiple hypothesis testing using the Benjamini-Hochberg method. Genes with at least one time point having a with a false-discovery rate of <0.10 as compared to the 0 hour expression for either anterior- or posterior-facing wound sites, as appropriate, were selected for further investigation. GeneCluster3.0 and Java TreeView were used for hierarchical clustering and visualization. Microarray data have been deposited at NCBI with accession number GSE56178. For analysis of progenitor genes in *zic-1* RNAi worms (Fig. 7A), neoblasts were isolated in similar way from animal fragments generated by head and tail removal (“trunks”) by FACS in a time series using a BD FacsAria SORP 5-Laser. RNA was extracted using RNeasy Mini Kit (Qiagen, 74104) and gene expression analyzed by RT-qPCR.

### Cloning

Array oligonucleotide sequences matching clone BPKG17485 [74] were blasted to a planarian transcriptome database (PlanMine, http://planmine.mpi-cbg.de) identifying exact matches for dd_Smed_v4_22585_0_1 and uc_Smed_v1_Contig48469. The predicted ORF encoded a protein of 529 amino acids with 87% identity to *Dugesia japonica zic-A* and identical to *Schmidtea mediterranea zicA* as named previously in a phylogenetic study [45], and we renamed this gene *zic-1*. *zic-2* was identified through a transcriptome search of Zic-family orthologs as dd_Smed_v4_15531_0_1 (PlanMine). The predicted ORF encoded a protein of 478 amino acids with 86% identity to *Dugesia japonica zicB* and identical to *Schmidtea mediterranea zicB* in a previous phylogenetic study [45].

Riboprobes for *zic-1* were made from a PCR product cloned by RT-PCR into pGEM vector using the primers 5′-CACTGCATGTATCAACACCAAG-3′ and 5′-AAGCAATTCTCCCACCGTTA-3′.

Double-stranded RNA (dsRNA) was generated by in vitro transcription or expression from a bacterial dsRNA expression vector (pPR244) as previously described [75]. Unless otherwise noted, all *zic-1* RNAi experiments used dsRNA derived from a 1611-bp *zic-1* fragment cloned using the primers 5′-CACTGCATGTATCAACACCAAG-3′ and 5′-AAGCAATTCTCCCACCGTTA-3′. Additionally, non-overlapping *zic-1* fragments were cloned to create a ∼700 bp 5′ fragment (using primers 5′-AAGCAATTCTCCCACCGTTA-3′ and 5′-AACCATTTCAATGCCCTTTC-3′) and a ∼900 bp 3′ fragment (using primers 5′-CGCGGACAATCTTTCCAATA-3′ and 5′-CACTGCATGTATCAACACCAAG-3′). *zic-2* was cloned using the primers 5′-TCACGGAATCTGAATGTGGA-3′ and 5′-TGAAACCGAGAGGTTTTCGT-3′.

Other riboprobes and/or dsRNAs (*smedwi-1, notum, beta-catenin-1, APC, wnt1, wntP-2, gpas, chat, sFRP-1, fzd-4, prep, pbx, pitx, follistatin, foxD, ovo, ap2, runt-1, slit, collagen, prog-1, ap2*) were as previously described [9], [14], [37]–[39], [41], [51], [56].

### Fixations and whole-mount *in situ* hybridization

Animals were fixed and stained as previously described [76]. In brief, animals were killed in 5% N-acetyl-cysteine in 1×PBS for 5 minutes and then fixed in formaldehyde for 17 minutes at room temperature. Subsequently, animals were bleached overnight (∼16 hours) in 6% hydrogen peroxide in methanol on a light box. Riboprobes were synthesized as previously described as digoxigenin- or fluorescein-labeled [76]. Colorimetric (NBT/BCIP) or fluorescence in situ hybridizations were performed as previously described [76]. For FISH, blocking solution was modified to MABT with 10% horse serum and 10% western blot blocking reagent (Roche) [77]. Anti-Dig-HRP and anti-FL-HRP antibodies were used at a 1∶2000 dilution, and anti-Dig-AP was used at a 1∶4000 dilution. Hoechst 33342 (Invitrogen) was used 1∶500 as counterstain. Images of colorimetric staining were acquired using a Leica M210F scope with a Leica DFC295 camera and adjusted for brightness and contrast. Fluorescence imaging was performed on either a Leica DM5500B compound microscope with Optigrid structured illumination system for optical sectioning or a Leica laser scanning SP5 confocal microscope at 40× or 63×. Images are maximum projections of a z-series with adjustments to brightness and contrast using ImageJ and Photoshop.

In FISH staining, cells expressing one or two genes of interest were manually counted from z-stacks using a ∼300 micron region near injury sites, each cell was labeled with color-coded marks and double checked by comparing with neighbor planes using ImageJ and Metamorph. For enumeration of WISH staining, NBT-BCIP precipitated material with the size and shape of cells were counted as the sum of ventral and dorsal views using a ∼300 micron region near injury sites using a dissecting microscope at 12× and additional magnification with a 1.7× turret dorsal and a manual counter. As a measure of the reproducibility of this assay, the signal to noise ratio (average/standard deviation) from 5 technical replicates scored on 3 specimens each was determined to be 9.8+/−2.3%.

### BrdU labeling and immunofluorescence

Uninjured worms were injected a solution of 5 mg/ml BrdU (Sigma-Aldrich/Fluka 16880) dissolved in water, two days prior to amputation of heads. Animals were fixed using NAC/FA solutions and rehydrated in a methanol series before FISH staining. Post-fixation in 4% formaldehyde and acid hydrolyzation in 2N HCl were performed after FISH detection, followed by antibody labeling of incorporated BrdU. Animals were blocked in PBSTB (PBSTx+0.25% BSA) for 4–6 hours at room temperature and incubated overnight in rat anti-BrdU (Oxford Biotechnology OBT0030S) antibody diluted 1∶1000 in PBSTB. Animals were rinsed in PBSTB and incubated overnight in goat anti-rat-HRP (AbCam ab7097) antibody diluted 1∶1000 in PBSTB blocking solution. Tyramide development was performed at room temperature for 1 hour in 1∶150 red tyramide (Alexa Fluor 568 T20914) in amplification buffer. BrdU imaging was performed on a Leica SP5 Confocal microscope. Polyclonal rabbit anti-SMEDWI-1 antibody (a kind gift of P. Reddien) was diluted 1∶2000 in PBSTB and detected by tyramide amplification as above [7].

### RNAi protocols

RNAi treatment was performed either by injection or by feeding. For RNAi by injection, dsRNA was synthesized from in vitro transcription reactions (Promega) using PCR templates with flanking T7 sequences (Denville), purified by phenol extraction and ethanol precipitation, resuspended in RNase-free water and annealed at 65°C for 10 minutes, 37°C for 20–30 minutes, then on ice for at least 10 minutes. dsRNA corresponding to *C. elegans unc-22*, a gene not present in the planarian genome, served as a negative control.

RNAi by injection was performed similarly to that described previously [37] and was performed as follows. Unless otherwise noted, animal fragments generated by head and tail removal (“trunks”) were injected 2–3 times with 30 nL of 3 µg/µl dsRNA and injected again the following two days, the animals were amputated anteriorly and posteriorly to remove ∼300 microns of tissue near the prior injury site, allowed to regenerate 6–8 days prior to another dsRNA injection and amputation of heads and tails for subsequent assaying of regeneration phenotypes and histological analysis.

For double-RNAi experiments, a similar injection strategy was used that involved three consecutive days of dsRNA injection, amputation and regeneration for 8 days, another three consecutive days of injections, then amputation of heads and tails and scoring of regeneration phenotypes 8 days later. dsRNA concentrations were normalized using control dsRNA so that every animal received an equivalent dose of *zic-1* and/or *beta-catenin-1*or *zic-2* dsRNA. For inhibition of *beta-catenin-1*, animals were given control dsRNA in the first set of dsRNA injections and *beta-catenin-1* dsRNA during the second set of injections. To generate *zic-1(RNAi);beta-catenin-1(RNAi)* animals, worms were given *zic-1* dsRNA during the first set of injections and a 1∶1 mixture of *beta-catenin-1* and *zic-1* dsRNA during the second set of injections. For inhibition of *zic-2*, animals were given a 1∶1 mixture of *zic-2* and control dsRNA in both sets of dsRNA injections. To generate *zic-1(RNAi);zic-2(RNAi)* animals, worms were given a 1∶1 mixture of both *zic-1* and *zic-2* dsRNA.

For experiments involving RNAi by bacterial feeding, animals were fed a mixture of liver paste and *E. coli* expressing dsRNA, as described previously [14], two times a week (every 3–4 days) for either 10 weeks without injury (Fig. S6), or 2 weeks (Fig. 5 and S7) prior to amputation of heads and tails.

### Realtime PCR

mRNA of *zic-1* was detected by realtime PCR using SYBR Green PCR Master Mix (Applied Biosystems). Total RNA was extracted by mechanical homogenization in Trizol (Invitrogen) from three regenerating fragments in three (Fig. 6C, S5E, and S9B) or four (Fig. 5C, S5E, and S8) biological replicates. RNA samples were DNase-treated (TURBO DNase, Ambion) and cDNA was synthesized using SuperScript III reverse transcriptase (Invitrogen). *zic-1* mRNA was detected using the following primers: 5′-TGGAAATAGAAATCTTGGTGGATT-3′ and 5′-AATCGGTTGTAATAGATTCGATGG-3′, *zic-2* mRNA was detected using 5′-CCTATGGTTGGATAAACACATGAA-3′ and 5′-CGACATGATCAAGTGTTAAGTGGT-3′, and *gapdh* mRNA was detected using previously described primers [14]. Primer sequences used in Fig. 1D and Fig. 7A are described in Table S3. Relative mRNA abundance was calculated using the delta-Ct method after verification of primer amplification efficiency. p-values below 0.05 were considered as significant differences.

## Supporting Information

Figure S1 Neoblasts are eliminated by gamma irradiation. In situ hybridizations to detect *smedwi-1* expression in animals untreated or irradiated (6000 Rads) 48 hours prior to amputation of heads and tails and fixation. These treatments eliminated *smedwi-1+* neoblasts (5/5 animals). Cartoon shows surgery and enlarged region. Anterior, top. Bars, 300 microns.
(TIF)

Figure S2 Expression profiling to identify genes activated in X1 neoblasts during head or tail regeneration. (A) Representative plots showing flow cytometry to isolate X1 neoblasts from macerated tissue fragments obtained from regions near injury sites in a time series after head or tail amputation. (B) Heat map showing log_2_ fold-change in gene expression in sorted X1 neoblasts at indicated times (hours) of anterior (A) or posterior (P) regeneration. Custom microarrays (Agilent) were probed by single-color labeling and in biological triplicate. Quantile array normalization and significance testing was performed in limma to compare neoblast gene expression at each time of regeneration with regionally-matched tissues obtained 5 minutes after surgery. Array features targeting 66 unique gene fragments (out of 58048 gene fragments probed) had altered expression at least one time across the series with a false-discovery rate significance threshold of 10% (Benjamini-Hochberg method). Gene annotation information shows identity of gene fragments identified by previous transcriptome assembly efforts [74] and *S. mediterranea* gene names where available, the identity of a top blastx match from a search against the human proteome (e-value<0.001), “no match” to describe gene fragments that do not fit these criteria. 42 of these genes were downregulated in neoblasts during regeneration (blue) whereas 24 of these genes were upregulated in neoblasts (yellow) either jointly or individually in anterior and posterior regeneration.
(TIF)

Figure S3 *notum+* cells of the regenerating anterior pole co-express SMEDWI-1 and *collagen* but not *prog-1*. Double FISH or FISH/immunostaining to detect expression of *notum* (magenta) and SMEDWI-1 protein, *prog-1* or *collagen* (green). Hoechst counterstain (gray). SMEDWI-1 protein is present in a broad population of cells recently derived from *smedwi-1+* neoblasts (86.5±8.3% *notum+* cells co-expressed SMEDWI-1, n = 5 animals). *prog-1* marks a population of post-mitotic neoblast descendants (no *notum+* cell co-expressed *prog-1*, n = 4 animals). *collagen* marks cells of the body wall musculature that produce positional cues important for regeneration (17.3±16.8% *notum+* cells co-expressed *collagen*, n = 5 animals). These results suggest that pole cells include *collagen*+ body wall musculature recently formed by neoblast differentiation. Red boxes indicate zoomed areas; yellow arrows, co-expression; white arrows, *notum+* only. Cartoon shows surgery and enlarged area. Anterior, top. Bars 75 microns (left) or 30 microns (right).
(TIF)

Figure S4 Additional histological analysis of *zic-1* RNAi animals. (A–D) In situ hybridizations of control or *zic-1(RNAi)* animals fixed after 8 days of regeneration and phenotypic scoring. (A) *zic-1(RNAi)* animals that regenerated eyeless anterior blastemas animals lack expression of *fzd4* (4/4 animals probed) and *wnt1* (9/9 animals probed) in their anterior, indicating these blastemas are not tails. (B) *pbx* is broadly expressed in both control and *zic-1(RNAi)* headless animals (5/5 animals probed). (C) Phenotypic scoring of animals treated with *zic-1* dsRNA alone (n = 56 animals) or diluted with an equal amount of control dsRNA (n = 37 animals). Dilution of *zic-1* dsRNA resulted in a higher frequency of cyclopia and a lower frequency of heedlessness, indicating cyclopia is a hypomorphic defect. Table shows rounded % penetrance for each phenotype. (D) *zic-1(RNAi)* cycloptic animals weakly expressed *notum* (9/9 animals) and *sFRP-1* (6/7 animals) in the anterior pole. These genes were not expressed in *zic-1(RNAi)* headless worms. Control animals are enlargements of those in Fig. 4 included for comparison. Cartoons show surgeries and enlarged areas. Anterior, top. Bars, 100 microns (A, D) or 300 microns (B).
(TIF)

Figure S5 *zic-2* is required for robust anterior regeneration patterning. *zic-2* is another Zic-family transcription factor present in the *S. mediterranea* genome. (A) in situ hybridization to detect expression of *zic-2* in intact animals. *zic-2* was expressed in the head region. Bar, 500 microns. (B) Double FISH to detect expression of *zic-2* and *smedwi-1* at 24 or 72 hours of regeneration in untreated or irradiated animals. 24-hour *zic-2* expression (magenta) was irradiation-sensitive and present in a subpopulation of *smedwi-1+* neoblast near the injury site. By 72 hours, *zic-2* expression was present in non-neoblast cells in the anterior region in an irradiation-sensitive manner. Yellow arrows, co-expressing cells. White arrows, not co-expressing cells. (C) Left, live animals regenerated with anterior defects after either *zic-2* RNAi or simultaneous inhibition of both *zic-1* and *zic-2* by RNAi. Table, scoring of anterior defects due single or double inhibition of *zic-1* or *zic-2* as percent penetrance (control, n = 9 animals; *zic-1* RNAi, n = 9 animals; *zic-2* RNAi, n = 11 animals; *zic-1(RNAi);zic-2(RNAi)*, n = 10 animals). Control dsRNA was added in single gene inhibitions to deliver an equivalent dose of dsRNA across indicated conditions. *zic-2* inhibition alone caused weakly penetrant cyclopia, and simultaneous inhibition of *zic-1* enhanced the frequency of head regeneration failure (headless animals) as compared to *zic-1* or *zic-2* inhibition alone (p-value<0.001 both tests, Fisher's exact test). (D) *zic-2* RNAi reduced but did not eliminate expression of *zic-1* at anterior-facing wounds 24 hours after injury (arrow). Right, quantitation of number of *zic-1+* cells in control or *zic-2(RNAi)* animals (n = 6 animals each condition). Error bars standard deviations, asterisks p-value<0.05 by t-test. (E) qPCR analysis of *zic-1* mRNA levels in *zic-2(RNAi)* worms (left), and *zic-2* mRNA in *zic-1(RNAi)* worms (right). Anterior, top. Scale bars, 75 microns (B), 250 microns (D), 300 microns (A, C).
(TIF)

Figure S6 Analysis of *zic-1* function in uninjured animals. (A) Animals fed bacteria expressing *zic-1* or control dsRNA twice a week for 60 days appeared normal. (B) After 5 weeks of RNAi, animals were fixed and stained for *sFRP-1* or *notum* expression. *zic-1(RNAi)* animals treated in this way had apparently normal expression of *sFRP-1* (3/3 animals probed). By contrast, RNAi of *zic-1* for 5 weeks in the absence of injury reduced but did not eliminate expression of *notum* at the anterior pole (5.8±0.5 cells, arrow, n = 4 animals) compared to control animals (11±1 cells, n = 3 animals), whereas *notum* expression at the anterior commissure in the region between the eyes was normal (10.7±2.3 cells in control animals versus 13±1.6 cells in *zic-1(RNAi)* animals, p-value = 0.221). Histogram shows quantification of cell number. Error bars standard deviations, asterisk indicates p<0.005 by t-test. Anterior, top. Scale bars, 150 microns (B) 300 microns (A).
(TIF)

Figure S7 Wnt signaling perturbation alters injury-induced expression of *zic-1* differentially across the A-P axis. (A–B) *beta-catenin-1* or *APC* were inhibited for two weeks by feeding as in Fig. 5A. Heads and tail fragments were fixed 24 hours after amputation and probed for *zic-1* expression. *APC* inhibition decreased *zic-1* expression (A) at posterior-facing wounds generated in anterior animal regions (5 fragments probed) and (B) at anterior-facing wounds generated in posterior animal regions (3 animals probed). By contrast, beta*-catenin-1* inhibition increased *zic-1* cell numbers in (B) anterior-facing wounds from posterior regions (6 fragments probed) and not (A) posterior-facing wounds from anterior regions (5 fragments probed). Right, numbers of *zic-1*+ cells under the indicated conditions. Asterisks, p-value<0.05 by t-test. (C) *zic-1* expression is not induced in the pre-pharyngeal or post-pharyngeal regions (arrows) of uninjured animals from the same cohort (4 animals probed each). Scale bars, 250 microns (A,B) or 300 microns (C).
(TIF)

Figure S8 Analysis of genes required for head regeneration and patterning for functions in injury-induced *zic-1* expression. (A) *patched* or *hedgehog* RNAi did not alter *zic-1* expression. Histograms show quantification by manual scoring of *zic-1*-expressing cells in anterior-facing wound sites of *patched* and *hedgehog* RNAi animals (left) and qPCR quantification of *zic-1* mRNA levels (right). (B) *pbx(RNAi)* and *follistatin(RNAi)* animals had reduced *zic-1*-expressing cell numbers (3 experiments), whereas inhibition of *prep*, *foxD*, *pitx* did not have an apparent effect. *zic-1+* cell number was quantified by manual scoring, shown in histogram. In A–B, worms of the same cohort displayed phenotypes as reported previously (*ptc* RNAi, 2/7 headless, 3/7 anterior tail; *prep* RNAi, 3/7 headless, 3/7 no eyes, 1/7 cycloptic; *pbx* RNAi, 6/6 no anterior or posterior regeneration; *foxD* RNAi 3/7 headless, 1/7 no eyes, 2/7 cycloptic; *follistatin* RNAi, 5/7 headless; *pitx* RNAi, 6/8 collapsed eyes). (C) Quantitation of *zic-1* expression by qPCR due to inhibition of *prep*, *pbx*, *follistatin*, *foxD* and *pitx* 24 hours after head and tail amputation using the Ct method for analysis, *gapdh* as a normalizing control, and four biological replicates of three animals each. *zic-1* expression was reduced in *pbx(RNAi)* and *pitx(RNAi)* animals (p-value<0.05 t-test), whereas it was not reduced due to *prep* RNAi. *follistatin* or *foxD* inhibition reduced *zic-1* expression although not significantly by a t-test (p-value>0.05, n.s.). We conclude that *pbx* is required for robust *zic-1* expression, and *zic-1* expression was still abundant in animals after inhibition of *prep*, *follistatin*, *foxD*, *hedgehog* or *patched*. Cartoons show surgery procedures and enlarged regions. Anterior, top. Scale bars, 300 microns.
(TIF)

Figure S9 Controls for double RNAi experiment. (A) Single and double-RNAi as indicated to examine interactions between *zic-1* and *beta-catenin-1* and animals were stained using in situ hybridizations for expression of *notum* at 72 hours and 14 days of regeneration. *beta-catenin-1* inhibition resulted in head outgrowth in the anterior and reduced *notum* expression at the anterior pole at 72 hours (5/5 animals) and 14 days (6/6 animals). *zic-1* RNAi eliminated head outgrowth and *notum* expression at the pole at both 72 hours (2/3 worms had no expression, 1/3 had weak expression) and 14 days (4/4 worms had no expression). Simultaneous inhibition of *beta-catenin-1* and *zic-1* prevented early 72-hour expression of *notum* at the anterior pole (5/5 animals). The heads that regenerate from such animals ultimately have reduced but not eliminated *notum* expression at their anterior pole by 14 days (9/9 animals). We conclude that experimental inhibition of *beta-catenin-1* likely can fulfill an early function of *zic-1* and *notum* important for head outgrowth, suggesting that the normal function of *zic-1* is to allow *notum* expression that inhibits *beta-catenin-1*. (B) qPCR to detect expression of *beta-catenin-1* in total RNA from control or *zic-1(RNAi)* animals purified 24 hours after head and tail amputation (3 biological replicates, 3 worms each). *beta-catenin-1* expression levels do not change due to *zic-1* RNAi (p-value>0.05, t-test, n.s. = not significant, *gapdh* as a normalizing control). Scale bars, 100 microns.
(TIF)

Figure S10 Analysis of neoblast defects in *zic-1(RNAi)* animals. (A) *zic-1(RNAi)* headless animals possess neoblasts throughout their bodies as visualized by in situ hybridization for *smedwi-1*. (B–E) In situ hybridizations to confirm select observations derived from qPCR expression analysis, Fig. 7A. (B) *zic-1* inhibition caused reduced expression of *ovo*, involved in early commitment of eye progenitor cells from neoblasts, by 48 hours after amputation. *zic-1(RNAi)* animals lacked *ovo* expression in the vicinity of the forming eyes (9/9 animals, arrow) compared to control animals (5/8) and also had reduced numbers of *ovo+* cells in neighboring regions (histogram shows number of *ovo+* cells, asterisk represents p<0.001, t-test). (C) *runt-1* expression occurred in *zic-1(RNAi)* animals. Graph shows quantitation of *runt-1+* cells at anterior- and posterior-facing wounds, with differences between *zic-1(RNAi)* and control worms not significant (n.s.) by t-test (p>0.05, at least 7 animals assayed in each condition). (D) *zic-1* expression was reduced but not eliminated after *runt-1* RNAi. Quantification of number of *zic-1+* cells in control and *runt-1(RNAi)* worms. (E) Right, *ap2* expression in intact animals. Left, *ap2* was expressed abundantly at the anterior-facing amputation sites of 48-hour regenerating tail fragments following *zic-1(RNAi)*. Of note, *ap2* is primarily activated at anterior-facing injury sites and therefore *zic-1* RNAi does not result in a wholesale failure of neoblast activities in the anterior. Cartoons show surgeries and enlarged areas. Anterior, top. Scale bars, 250 (B, C, D, E right) or 500 microns (A, E left).
(TIF)

Table S1 Expression data from Microarray and qPCR validation. Table showing gene expression information for as measured by microarray and qPCR (see Fig. S2B, Fig. 1B–D and text for details), with sequence ID, gene name, the highest match to the human proteome by blastx of the nucleotide sequence with corresponding e-value. Genes with e-value>0.001 and not previously assigned a name are listed as “novel”. See Figure S2B and Figure 1B–D for experimental details. Table shows log_2_ fold-changes for each gene as compared to the 0-hour timepoint for either anterior or posterior regeneration. Yellow indicates upregulation and blue indicates downregulation. Microarray q-values are p-values adjusted for multiple hypothesis testing using the Benjamini-Hochberg method using the limma package of Bioconductor implemented in R (see Methods), with q<0.10 the criteria for gene selection and indicated with red shading. Realtime PCR p-values are from a Student's t-test comparing expression at the indicated timepoint with expression at the 0-hour timepoint for that condition, with p<0.05 indicated in red. 21 genes with expression upregulated in regeneration and p<0.05 were considered validated and shown in Fig. 1C–D, whereas three genes (BPKG9013, BPKG24021 and BPKG40848) did not meet these criteria.
(XLSX)

Table S2 qPCR fold-change values in X1 cells after *zic-1* RNAi. qPCR fold-change and p-values associated with Figure 7A. Blue indicates downregulation, yellow upregulation. p-values<0.05 are highlighted in red.
(PDF)

Table S3 qPCR primers. Sequences of primers used in this study for qPCR.
(PDF)
