# Supplementary figures and images for: zic-1 Expression in Planarian Neoblasts after Injury Controls Anterior Pole Regeneration
Source: PLoS Genet. 2014 Jul 3;10(7):e1004452. doi: 10.1371/journal.pgen.1004452 (PMC4081000; doi:10.1371/journal.pgen.1004452)

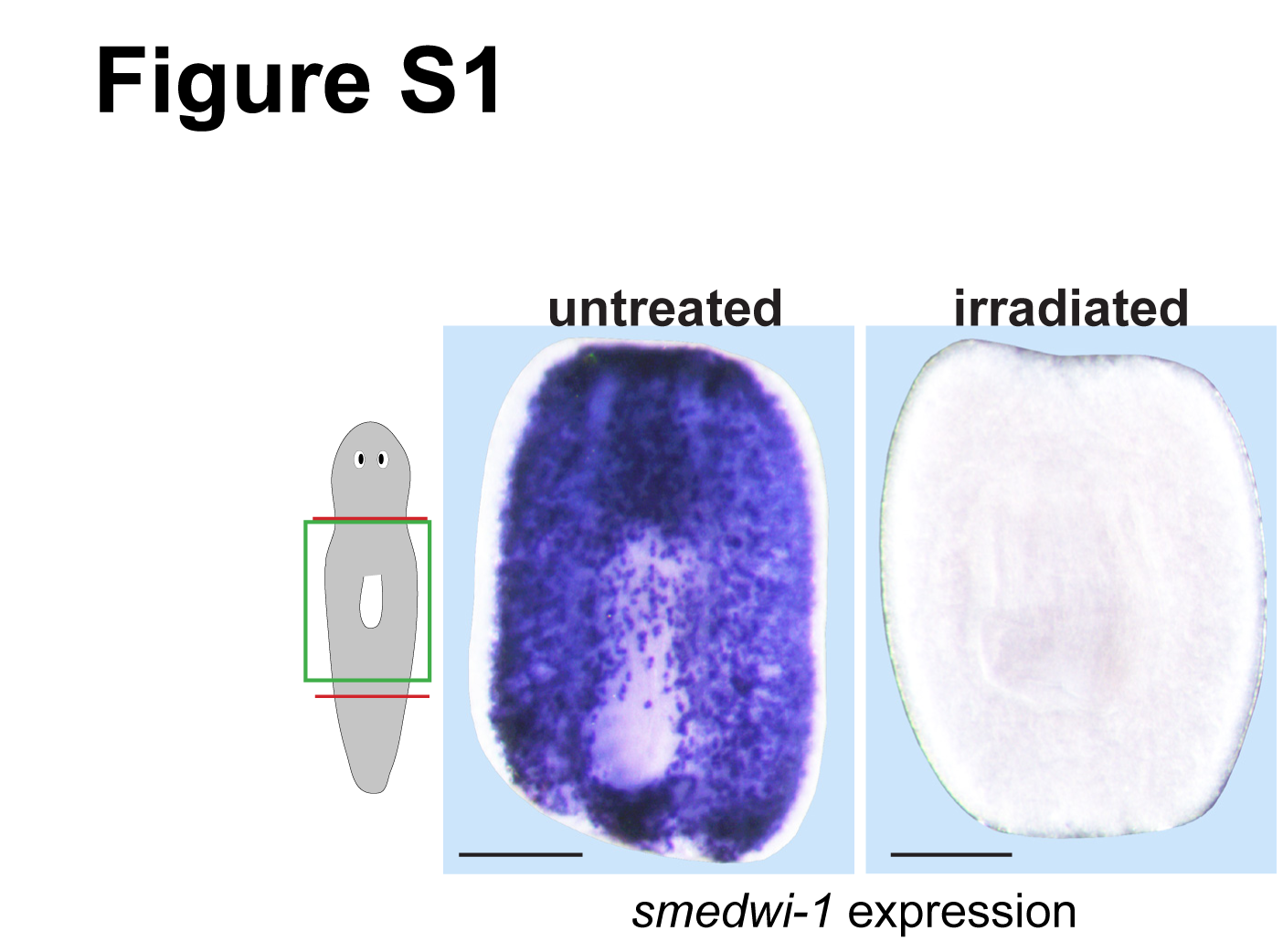

Supplement: Figure S1 — Neoblasts are eliminated by gamma irradiation. In situ hybridizations to detect smedwi-1 expression in animals untreated or irradiated (6000 Rads) 48 hours prior to amputation of heads and tails and fixation. These treatments eliminated smedwi-1+ neoblasts (5/5 animals). Cartoon shows surgery and enlarged region. Anterior, top. Bars, 300 microns. (TIF) [file pgen.1004452.s001.tif]

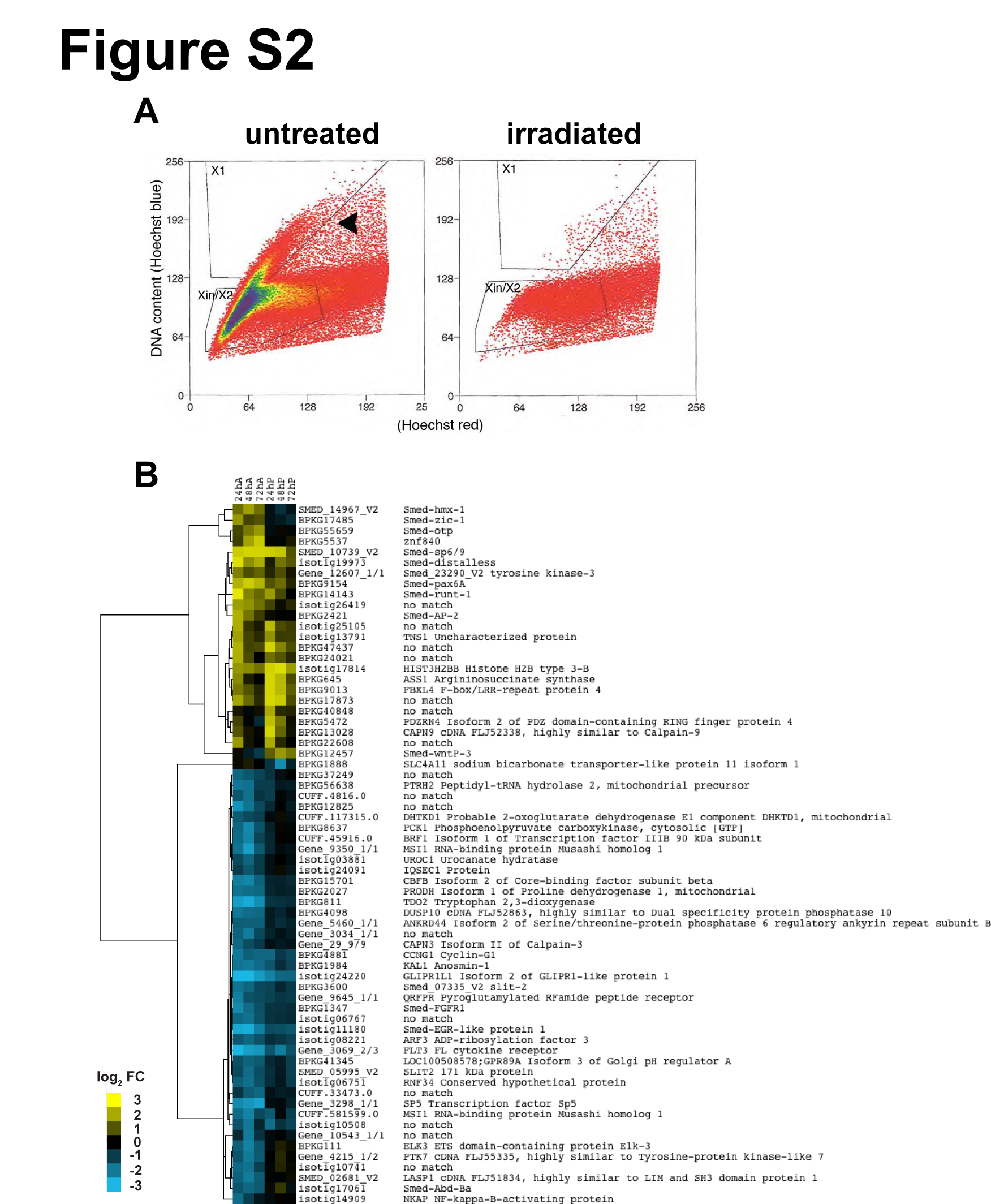

Supplement: Figure S2 — Expression profiling to identify genes activated in X1 neoblasts during head or tail regeneration. (A) Representative plots showing flow cytometry to isolate X1 neoblasts from macerated tissue fragments obtained from regions near injury sites in a time series after head or tail amputation. (B) Heat map showing log2 fold-change in gene expression in sorted X1 neoblasts at indicated times (hours) of anterior (A) or posterior (P) regeneration. Custom microarrays (Agilent) were probed by single-color labeling and in biological triplicate. Quantile array normalization and significance testing was performed in limma to compare neoblast gene expression at each time of regeneration with regionally-matched tissues obtained 5 minutes after surgery. Array features targeting 66 unique gene fragments (out of 58048 gene fragments probed) had altered expression at least one time across the series with a false-discovery rate significance threshold of 10% (Benjamini-Hochberg method). Gene annotation information shows identity of gene fragments identified by previous transcriptome assembly efforts [74] and S. mediterranea gene names where available, the identity of a top blastx match from a search against the human proteome (e-value<0.001), “no match” to describe gene fragments that do not fit these criteria. 42 of these genes were downregulated in neoblasts during regeneration (blue) whereas 24 of these genes were upregulated in neoblasts (yellow) either jointly or individually in anterior and posterior regeneration. (TIF) [file pgen.1004452.s002.tif]

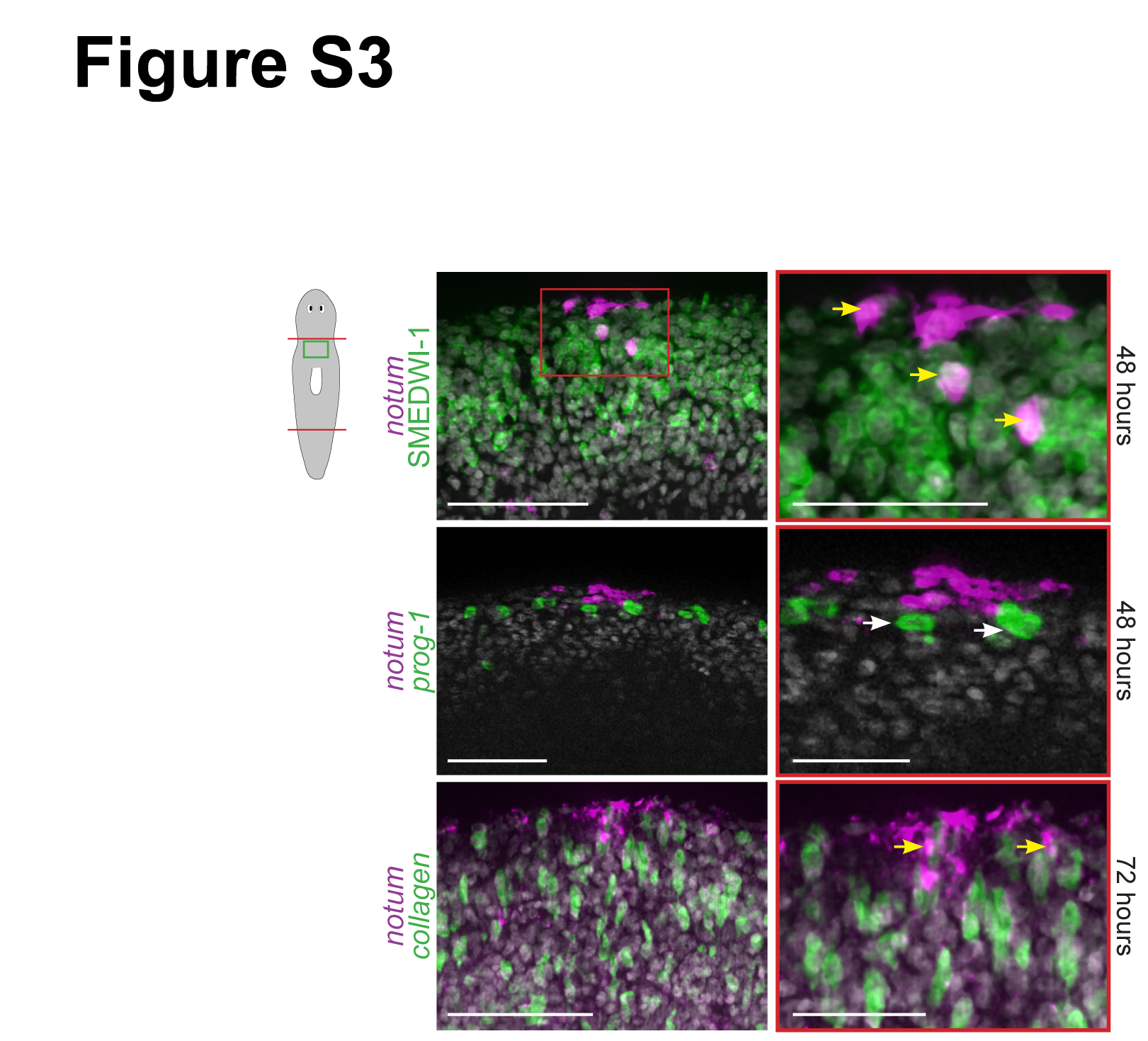

Supplement: Figure S3 — notum+ cells of the regenerating anterior pole co-express SMEDWI-1 and collagen but not prog-1. Double FISH or FISH/immunostaining to detect expression of notum (magenta) and SMEDWI-1 protein, prog-1 or collagen (green). Hoechst counterstain (gray). SMEDWI-1 protein is present in a broad population of cells recently derived from smedwi-1+ neoblasts (86.5±8.3% notum+ cells co-expressed SMEDWI-1, n = 5 animals). prog-1 marks a population of post-mitotic neoblast descendants (no notum+ cell co-expressed prog-1, n = 4 animals). collagen marks cells of the body wall musculature that produce positional cues important for regeneration (17.3±16.8% notum+ cells co-expressed collagen, n = 5 animals). These results suggest that pole cells include collagen+ body wall musculature recently formed by neoblast differentiation. Red boxes indicate zoomed areas; yellow arrows, co-expression; white arrows, notum+ only. Cartoon shows surgery and enlarged area. Anterior, top. Bars 75 microns (left) or 30 microns (right). (TIF) [file pgen.1004452.s003.tif]

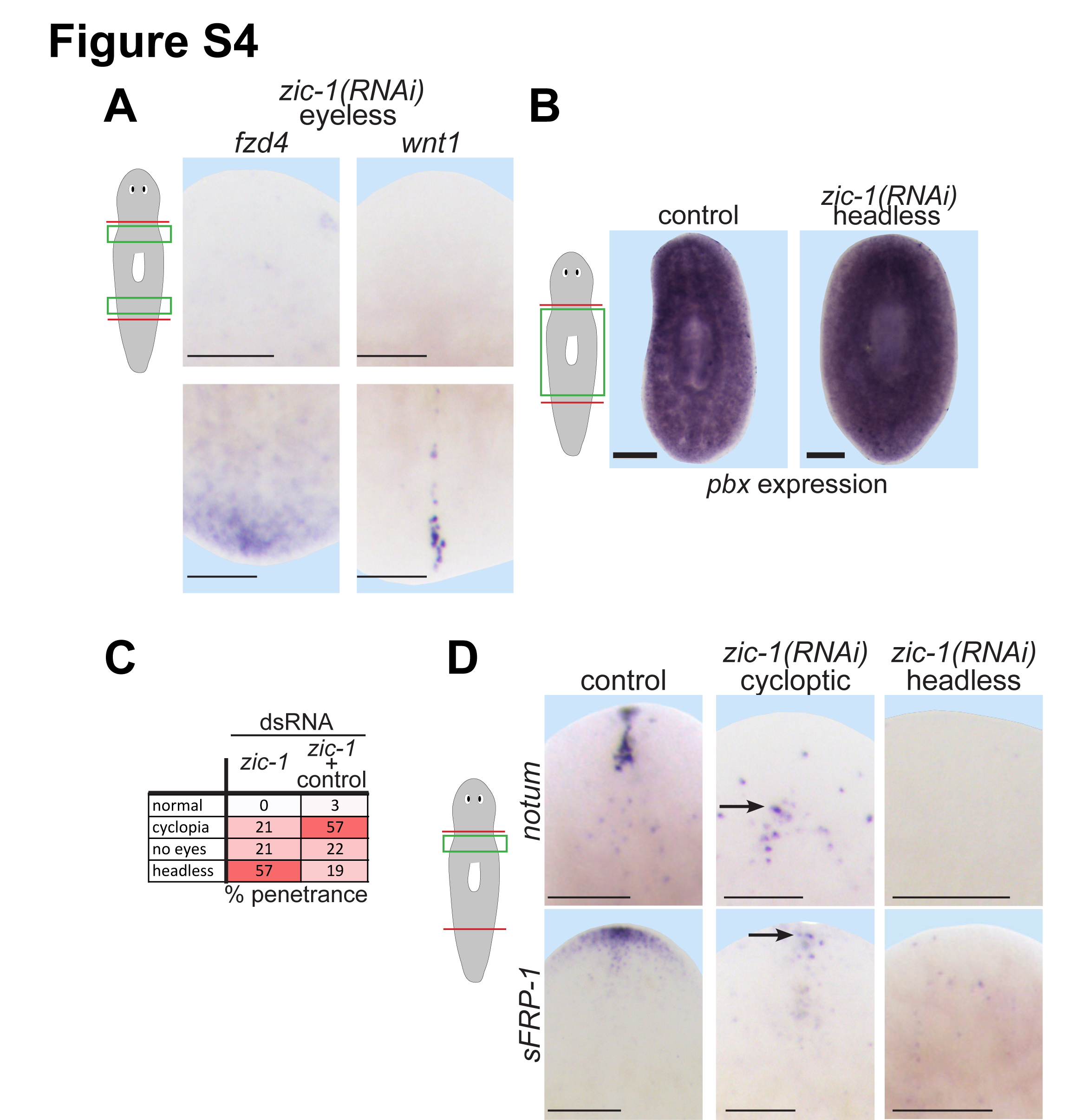

Supplement: Figure S4 — Additional histological analysis of zic-1 RNAi animals. (A–D) In situ hybridizations of control or zic-1(RNAi) animals fixed after 8 days of regeneration and phenotypic scoring. (A) zic-1(RNAi) animals that regenerated eyeless anterior blastemas animals lack expression of fzd4 (4/4 animals probed) and wnt1 (9/9 animals probed) in their anterior, indicating these blastemas are not tails. (B) pbx is broadly expressed in both control and zic-1(RNAi) headless animals (5/5 animals probed). (C) Phenotypic scoring of animals treated with zic-1 dsRNA alone (n = 56 animals) or diluted with an equal amount of control dsRNA (n = 37 animals). Dilution of zic-1 dsRNA resulted in a higher frequency of cyclopia and a lower frequency of heedlessness, indicating cyclopia is a hypomorphic defect. Table shows rounded % penetrance for each phenotype. (D) zic-1(RNAi) cycloptic animals weakly expressed notum (9/9 animals) and sFRP-1 (6/7 animals) in the anterior pole. These genes were not expressed in zic-1(RNAi) headless worms. Control animals are enlargements of those in Fig. 4 included for comparison. Cartoons show surgeries and enlarged areas. Anterior, top. Bars, 100 microns (A, D) or 300 microns (B). (TIF) [file pgen.1004452.s004.tif]

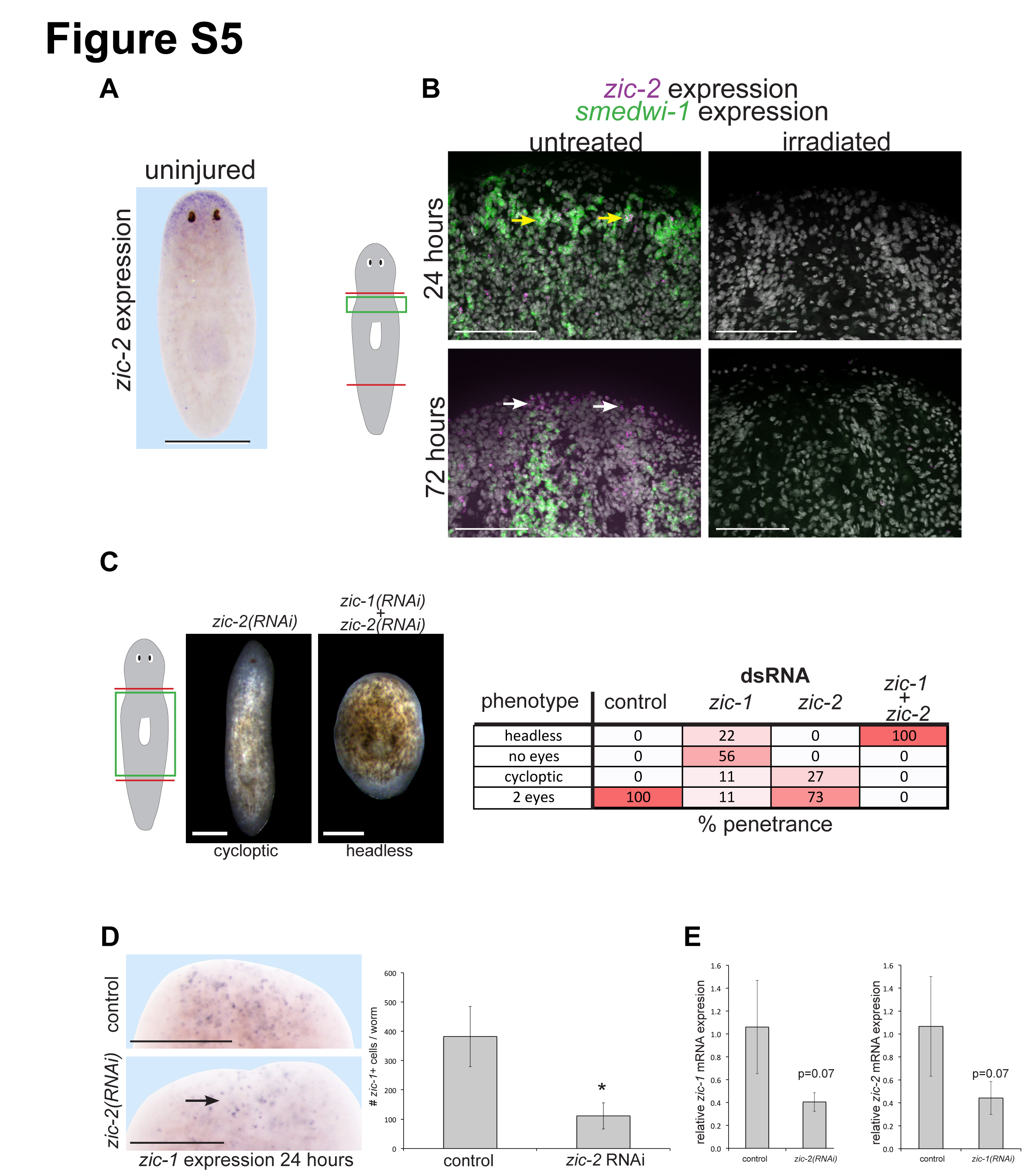

Supplement: Figure S5 — zic-2 is required for robust anterior regeneration patterning. zic-2 is another Zic-family transcription factor present in the S. mediterranea genome. (A) in situ hybridization to detect expression of zic-2 in intact animals. zic-2 was expressed in the head region. Bar, 500 microns. (B) Double FISH to detect expression of zic-2 and smedwi-1 at 24 or 72 hours of regeneration in untreated or irradiated animals. 24-hour zic-2 expression (magenta) was irradiation-sensitive and present in a subpopulation of smedwi-1+ neoblast near the injury site. By 72 hours, zic-2 expression was present in non-neoblast cells in the anterior region in an irradiation-sensitive manner. Yellow arrows, co-expressing cells. White arrows, not co-expressing cells. (C) Left, live animals regenerated with anterior defects after either zic-2 RNAi or simultaneous inhibition of both zic-1 and zic-2 by RNAi. Table, scoring of anterior defects due single or double inhibition of zic-1 or zic-2 as percent penetrance (control, n = 9 animals; zic-1 RNAi, n = 9 animals; zic-2 RNAi, n = 11 animals; zic-1(RNAi);zic-2(RNAi), n = 10 animals). Control dsRNA was added in single gene inhibitions to deliver an equivalent dose of dsRNA across indicated conditions. zic-2 inhibition alone caused weakly penetrant cyclopia, and simultaneous inhibition of zic-1 enhanced the frequency of head regeneration failure (headless animals) as compared to zic-1 or zic-2 inhibition alone (p-value<0.001 both tests, Fisher's exact test). (D) zic-2 RNAi reduced but did not eliminate expression of zic-1 at anterior-facing wounds 24 hours after injury (arrow). Right, quantitation of number of zic-1+ cells in control or zic-2(RNAi) animals (n = 6 animals each condition). Error bars standard deviations, asterisks p-value<0.05 by t-test. (E) qPCR analysis of zic-1 mRNA levels in zic-2(RNAi) worms (left), and zic-2 mRNA in zic-1(RNAi) worms (right). Anterior, top. Scale bars, 75 microns (B), 250 microns (D), 300 microns (A, C). (TIF) [file pgen.1004452.s005.tif]

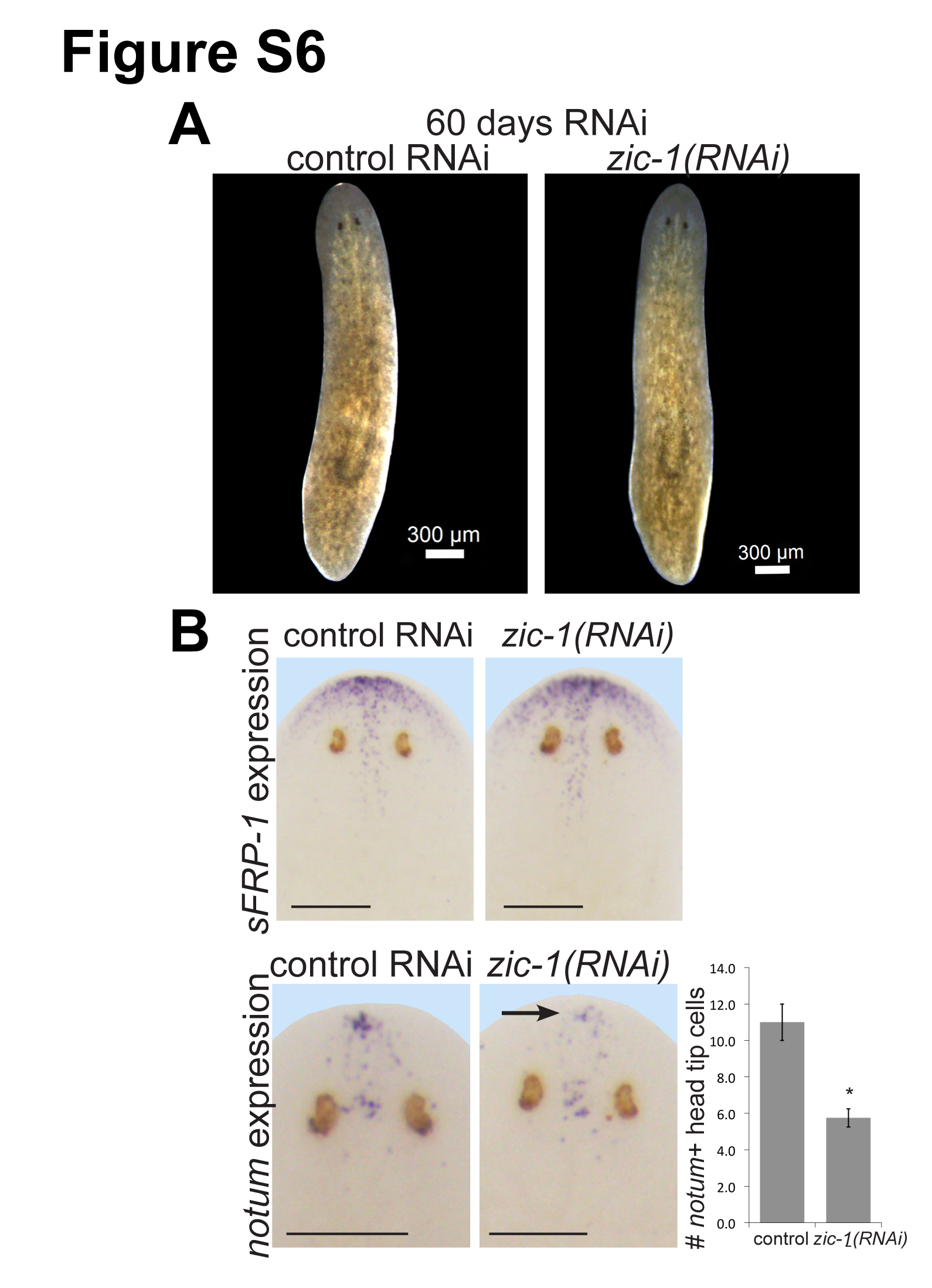

Supplement: Figure S6 — Analysis of zic-1 function in uninjured animals. (A) Animals fed bacteria expressing zic-1 or control dsRNA twice a week for 60 days appeared normal. (B) After 5 weeks of RNAi, animals were fixed and stained for sFRP-1 or notum expression. zic-1(RNAi) animals treated in this way had apparently normal expression of sFRP-1 (3/3 animals probed). By contrast, RNAi of zic-1 for 5 weeks in the absence of injury reduced but did not eliminate expression of notum at the anterior pole (5.8±0.5 cells, arrow, n = 4 animals) compared to control animals (11±1 cells, n = 3 animals), whereas notum expression at the anterior commissure in the region between the eyes was normal (10.7±2.3 cells in control animals versus 13±1.6 cells in zic-1(RNAi) animals, p-value = 0.221). Histogram shows quantification of cell number. Error bars standard deviations, asterisk indicates p<0.005 by t-test. Anterior, top. Scale bars, 150 microns (B) 300 microns (A). (TIF) [file pgen.1004452.s006.tif]

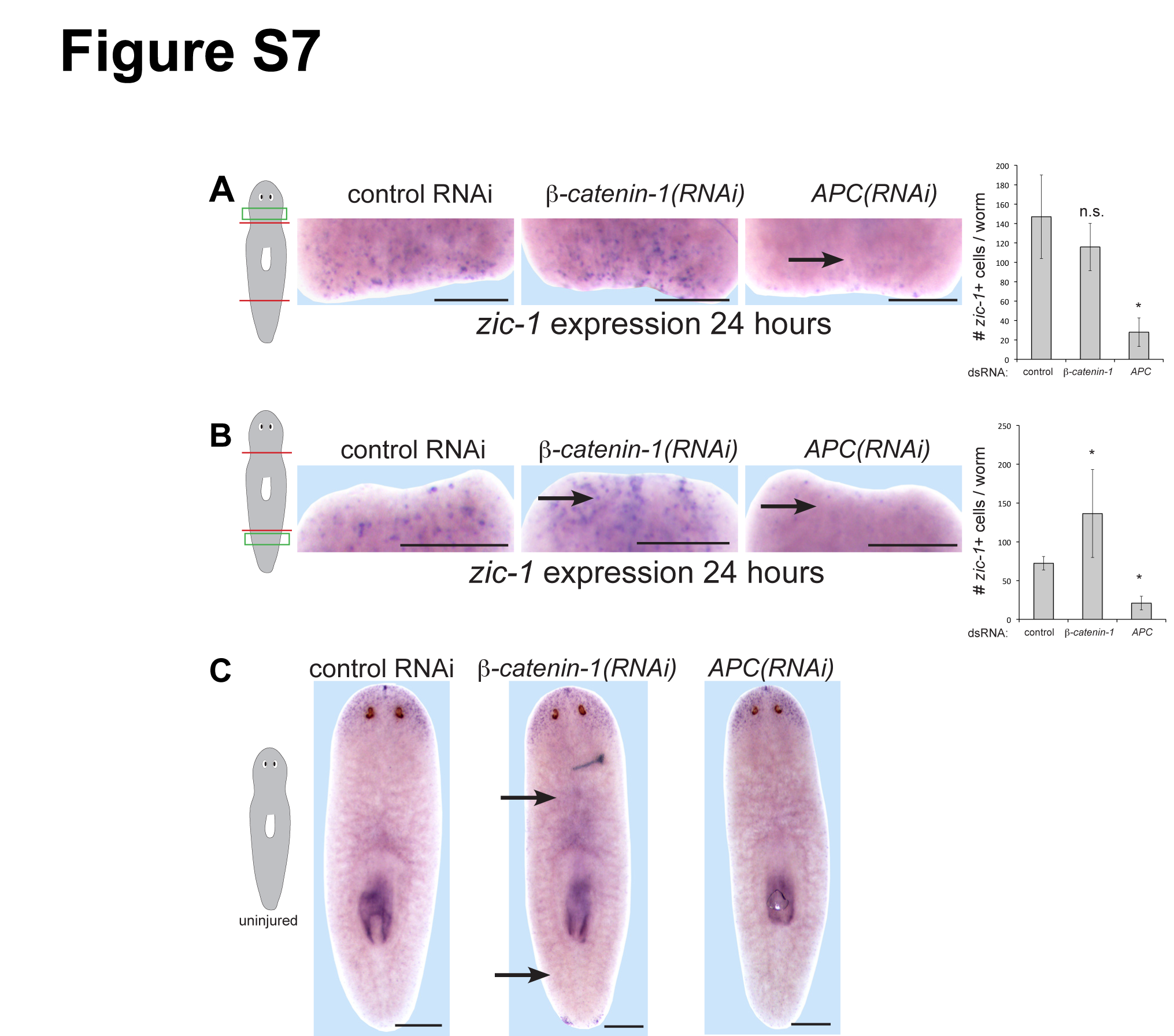

Supplement: Figure S7 — Wnt signaling perturbation alters injury-induced expression of zic-1 differentially across the A-P axis. (A–B) beta-catenin-1 or APC were inhibited for two weeks by feeding as in Fig. 5A. Heads and tail fragments were fixed 24 hours after amputation and probed for zic-1 expression. APC inhibition decreased zic-1 expression (A) at posterior-facing wounds generated in anterior animal regions (5 fragments probed) and (B) at anterior-facing wounds generated in posterior animal regions (3 animals probed). By contrast, beta-catenin-1 inhibition increased zic-1 cell numbers in (B) anterior-facing wounds from posterior regions (6 fragments probed) and not (A) posterior-facing wounds from anterior regions (5 fragments probed). Right, numbers of zic-1+ cells under the indicated conditions. Asterisks, p-value<0.05 by t-test. (C) zic-1 expression is not induced in the pre-pharyngeal or post-pharyngeal regions (arrows) of uninjured animals from the same cohort (4 animals probed each). Scale bars, 250 microns (A,B) or 300 microns (C). (TIF) [file pgen.1004452.s007.tif]

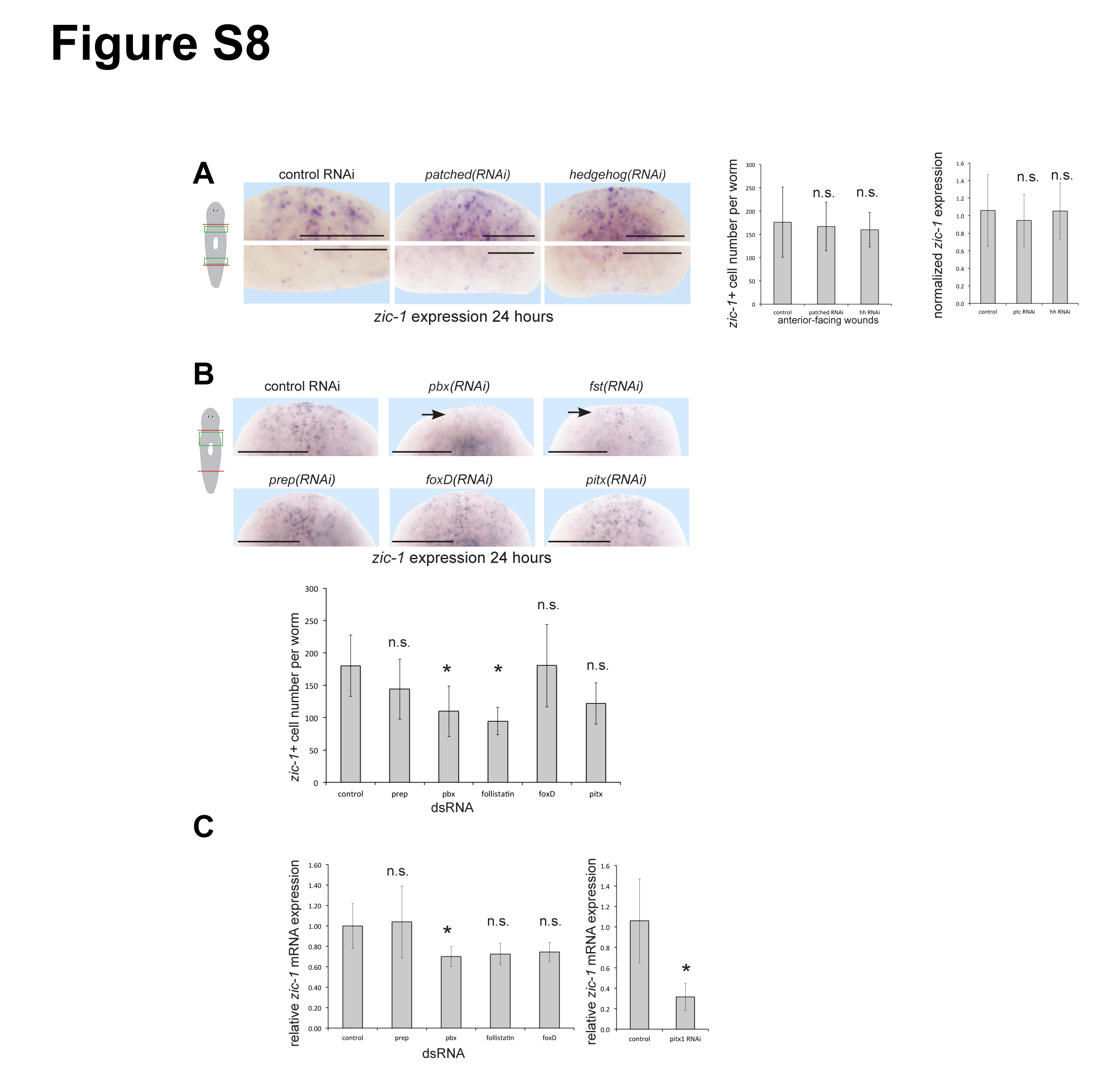

Supplement: Figure S8 — Analysis of genes required for head regeneration and patterning for functions in injury-induced zic-1 expression. (A) patched or hedgehog RNAi did not alter zic-1 expression. Histograms show quantification by manual scoring of zic-1-expressing cells in anterior-facing wound sites of patched and hedgehog RNAi animals (left) and qPCR quantification of zic-1 mRNA levels (right). (B) pbx(RNAi) and follistatin(RNAi) animals had reduced zic-1-expressing cell numbers (3 experiments), whereas inhibition of prep, foxD, pitx did not have an apparent effect. zic-1+ cell number was quantified by manual scoring, shown in histogram. In A–B, worms of the same cohort displayed phenotypes as reported previously (ptc RNAi, 2/7 headless, 3/7 anterior tail; prep RNAi, 3/7 headless, 3/7 no eyes, 1/7 cycloptic; pbx RNAi, 6/6 no anterior or posterior regeneration; foxD RNAi 3/7 headless, 1/7 no eyes, 2/7 cycloptic; follistatin RNAi, 5/7 headless; pitx RNAi, 6/8 collapsed eyes). (C) Quantitation of zic-1 expression by qPCR due to inhibition of prep, pbx, follistatin, foxD and pitx 24 hours after head and tail amputation using the Ct method for analysis, gapdh as a normalizing control, and four biological replicates of three animals each. zic-1 expression was reduced in pbx(RNAi) and pitx(RNAi) animals (p-value<0.05 t-test), whereas it was not reduced due to prep RNAi. follistatin or foxD inhibition reduced zic-1 expression although not significantly by a t-test (p-value>0.05, n.s.). We conclude that pbx is required for robust zic-1 expression, and zic-1 expression was still abundant in animals after inhibition of prep, follistatin, foxD, hedgehog or patched. Cartoons show surgery procedures and enlarged regions. Anterior, top. Scale bars, 300 microns. (TIF) [file pgen.1004452.s008.tif]

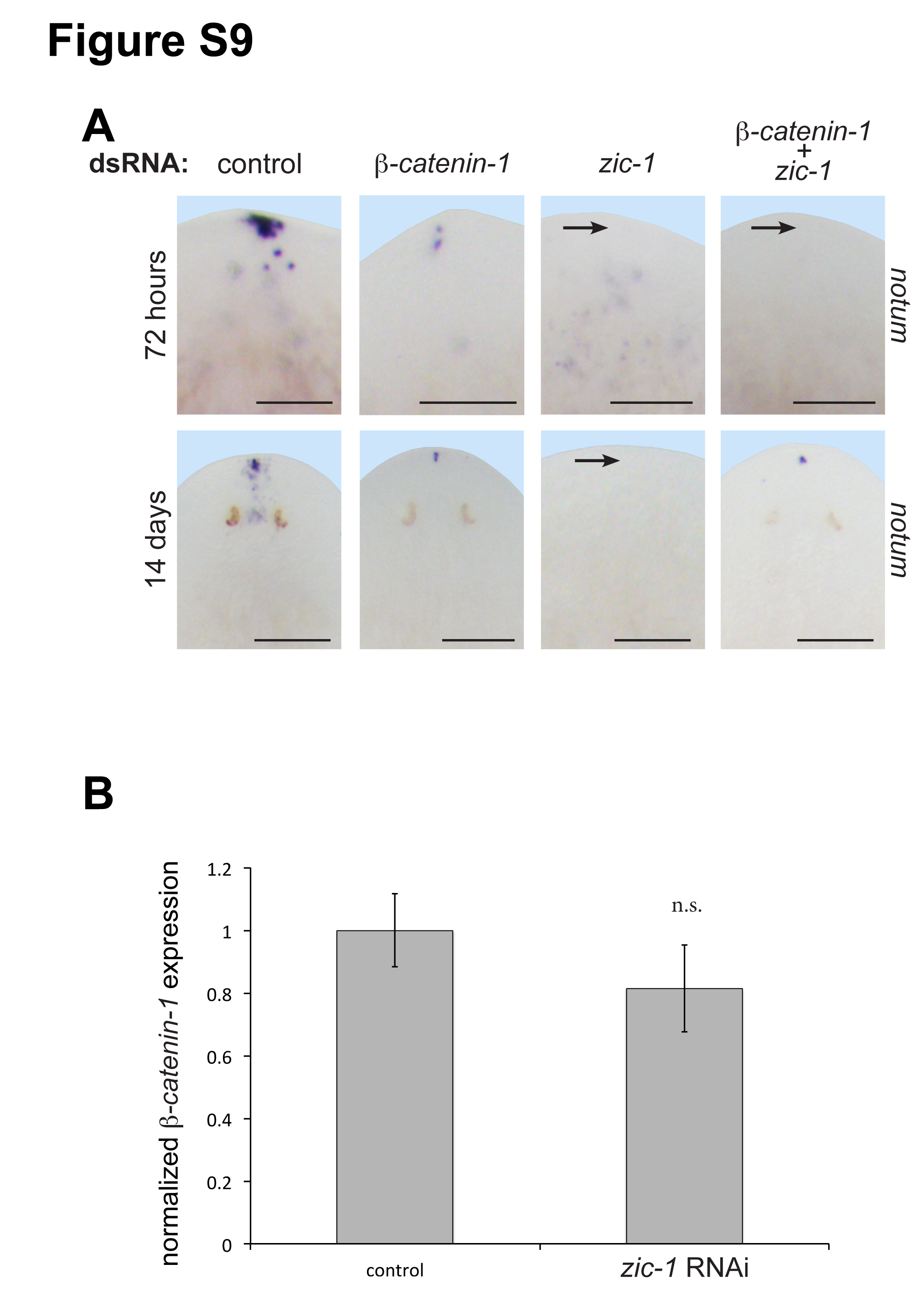

Supplement: Figure S9 — Controls for double RNAi experiment. (A) Single and double-RNAi as indicated to examine interactions between zic-1 and beta-catenin-1 and animals were stained using in situ hybridizations for expression of notum at 72 hours and 14 days of regeneration. beta-catenin-1 inhibition resulted in head outgrowth in the anterior and reduced notum expression at the anterior pole at 72 hours (5/5 animals) and 14 days (6/6 animals). zic-1 RNAi eliminated head outgrowth and notum expression at the pole at both 72 hours (2/3 worms had no expression, 1/3 had weak expression) and 14 days (4/4 worms had no expression). Simultaneous inhibition of beta-catenin-1 and zic-1 prevented early 72-hour expression of notum at the anterior pole (5/5 animals). The heads that regenerate from such animals ultimately have reduced but not eliminated notum expression at their anterior pole by 14 days (9/9 animals). We conclude that experimental inhibition of beta-catenin-1 likely can fulfill an early function of zic-1 and notum important for head outgrowth, suggesting that the normal function of zic-1 is to allow notum expression that inhibits beta-catenin-1. (B) qPCR to detect expression of beta-catenin-1 in total RNA from control or zic-1(RNAi) animals purified 24 hours after head and tail amputation (3 biological replicates, 3 worms each). beta-catenin-1 expression levels do not change due to zic-1 RNAi (p-value>0.05, t-test, n.s. = not significant, gapdh as a normalizing control). Scale bars, 100 microns. (TIF) [file pgen.1004452.s009.tif]

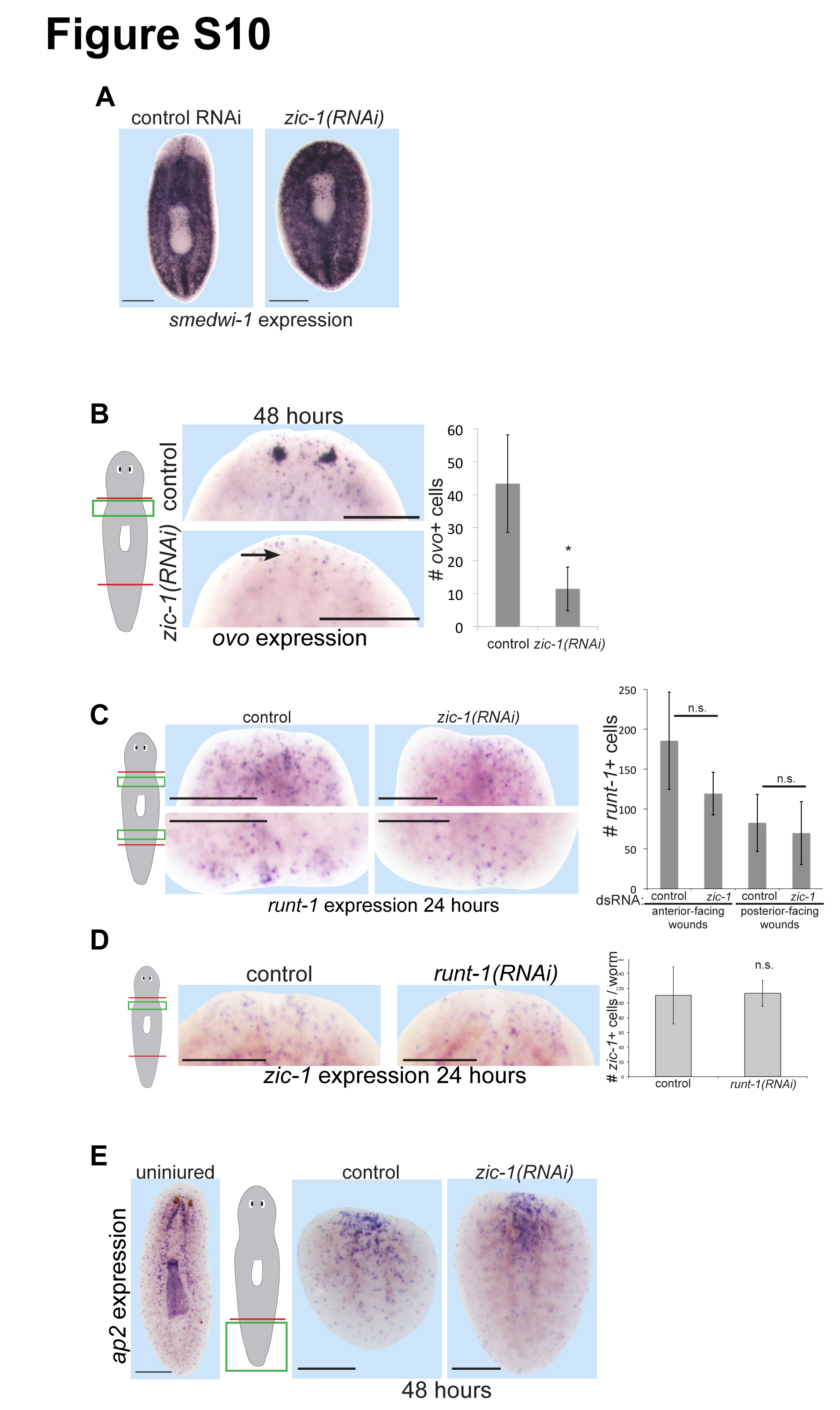

Supplement: Figure S10 — Analysis of neoblast defects in zic-1(RNAi) animals. (A) zic-1(RNAi) headless animals possess neoblasts throughout their bodies as visualized by in situ hybridization for smedwi-1. (B–E) In situ hybridizations to confirm select observations derived from qPCR expression analysis, Fig. 7A. (B) zic-1 inhibition caused reduced expression of ovo, involved in early commitment of eye progenitor cells from neoblasts, by 48 hours after amputation. zic-1(RNAi) animals lacked ovo expression in the vicinity of the forming eyes (9/9 animals, arrow) compared to control animals (5/8) and also had reduced numbers of ovo+ cells in neighboring regions (histogram shows number of ovo+ cells, asterisk represents p<0.001, t-test). (C) runt-1 expression occurred in zic-1(RNAi) animals. Graph shows quantitation of runt-1+ cells at anterior- and posterior-facing wounds, with differences between zic-1(RNAi) and control worms not significant (n.s.) by t-test (p>0.05, at least 7 animals assayed in each condition). (D) zic-1 expression was reduced but not eliminated after runt-1 RNAi. Quantification of number of zic-1+ cells in control and runt-1(RNAi) worms. (E) Right, ap2 expression in intact animals. Left, ap2 was expressed abundantly at the anterior-facing amputation sites of 48-hour regenerating tail fragments following zic-1(RNAi). Of note, ap2 is primarily activated at anterior-facing injury sites and therefore zic-1 RNAi does not result in a wholesale failure of neoblast activities in the anterior. Cartoons show surgeries and enlarged areas. Anterior, top. Scale bars, 250 (B, C, D, E right) or 500 microns (A, E left). (TIF) [file pgen.1004452.s010.tif]
