# Supplementary material for: zic-1 Expression in Planarian Neoblasts after Injury Controls Anterior Pole Regeneration
Source: PLoS Genet. 2014 Jul 3;10(7):e1004452. doi: 10.1371/journal.pgen.1004452 (PMC4081000; doi:10.1371/journal.pgen.1004452)
Supplement: Table S2 — qPCR fold-change values in X1 cells after zic-1 RNAi. qPCR fold-change and p-values associated with Figure 7A. Blue indicates downregulation, yellow upregulation. p-values<0.05 are highlighted in red. (PDF) [file pgen.1004452.s012.pdf]

Table S2. qPCR fold-change values in X1 cells after zic-1 RNAi

	log2(FC) zic-1 RNAi vs control			p-value (2-tailed T-test)		
mRNA probed	0h	24h	48h	0h	24h	48h
ovo	-2.17	-1.40	-1.67	0.00158	0.02049	0.00182
zic	-1.85	-1.17	-2.24	0.00004	0.08325	0.09153
notum	-1.26	-0.19	-2.05	0.07840	0.20451	0.03705
hmx-1	-0.82	-1.72	-0.97	0.17517	0.10500	0.12510
dlx	-0.95	-1.13	-0.81	0.07484	0.00126	0.07144
sp6/9	-1.03	-0.05	-0.01	0.06196	0.76045	0.97402
OtxA	-0.61	-0.06	-0.05	0.11256	0.92091	0.91337
pax6A	-0.64	-0.10	-0.09	0.00665	0.65885	0.82223
soxB	-0.49	-0.10	0.12	0.00283	0.77651	0.80063
runt-1	-0.50	-0.19	0.29	0.04359	0.52839	0.22042
six1-2	-0.34	0.03	0.17	0.11294	0.94451	0.66348
six3-1	-0.62	0.04	0.39	0.02089	0.84524	0.20175
sim	-0.09	0.14	0.24	0.63697	0.65324	0.24277
coe	-0.04	0.21	0.40	0.81728	0.58962	0.62987
ap2	-0.28	-0.04	0.77	0.29528	0.92250	0.02467
