# Supplementary material for: zic-1 Expression in Planarian Neoblasts after Injury Controls Anterior Pole Regeneration
Source: PLoS Genet. 2014 Jul 3;10(7):e1004452. doi: 10.1371/journal.pgen.1004452 (PMC4081000; doi:10.1371/journal.pgen.1004452)
Supplement: Table S3 — qPCR primers. Sequences of primers used in this study for qPCR. (PDF) [file pgen.1004452.s013.pdf]

Table S3, primers used for realtime PCR

gene	left primer	right primer
Smed_coe	CTTCATCTGGTCACGGGAGT	CTTCATCTGGTCACGGGAGT
Smed_eya	GTCCACCAACAAATCCTTCG	GTCCACCAACAAATCCTTCG
Smed_hesl-3	GGCAGAGCTCAAAGAATTGG	GGCAGAGCTCAAAGAATTGG
Smed_otxA	GCAGCAAATCAACTGCAAAA	GCAGCAAATCAACTGCAAAA
Smed_pax6A	GGCAGCAAACCAAGAGTAGC	GGCAGCAAACCAAGAGTAGC
Smed_sim	AGGCATTCGCATCAAACTCT	AGGCATTCGCATCAAACTCT
Smed_six1-2	GATTTGGGACGGTGAAGAAA	GATTTGGGACGGTGAAGAAA
Smed_six3-1	GATAAATATCGTGTCCGGAAAAAG	GATAAATATCGTGTCCGGAAAAAG
Smed_soxB	TCAAGGCCCATTTCAATCTC	TCAAGGCCCATTTCAATCTC
Smed-ap2	CACAGATTTACTCAACAAGGATCG	CACAGATTTACTCAACAAGGATCG
Smed-distalless	CCAGTCGGGCTATCAGTACTATTT	CCAGTCGGGCTATCAGTACTATTT
Smed-otp	ACGATAAATCTTCACCGAACAAAT	ACGATAAATCTTCACCGAACAAAT
Smed-pax6A	GGTTCAAACGGAACAAGTTCTACT	GGTTCAAACGGAACAAGTTCTACT
Smed-runt-1	GAAGCAAGATTCAATGACTTGAGA	GAAGCAAGATTCAATGACTTGAGA
Smed-sp6-9	ACTTTCCAGTCTATTCCTGCAAAC	ACTTTCCAGTCTATTCCTGCAAAC
Smed-tyrosine kinase-3	ATTTATCCAGCATACGGAGACTTC	ATTTATCCAGCATACGGAGACTTC
Smed-wntP-3	GGAATACAGGAATGTCAACATCAA	GGAATACAGGAATGTCAACATCAA
Smed-zicA	AAATCTTGGTGGATTTTGCTATTC	AAATCTTGGTGGATTTTGCTATTC
ASS1	CTAGTCCTGACCAACCTGAGAAAT	CTAGTCCTGACCAACCTGAGAAAT
CAPN9	TAATGTTCGAAATTTGTCAAAGGA	TAATGTTCGAAATTTGTCAAAGGA
FBXL4	ATGTCTATTCGATTTTCGGGATTA	ATGTCTATTCGATTTTCGGGATTA
h2b	GTAAAGTAGCGTCAAAATCTGCAA	GTAAAGTAGCGTCAAAATCTGCAA
BPKG17873	CAGCAATACCCGATAATTATGACA	CAGCAATACCCGATAATTATGACA
BPKG22608	CAAACTAAAAAGCGTCATAGACCA	CAAACTAAAAAGCGTCATAGACCA
BPKG40848	TGCTTTATGGAAATAAACAGGTCA	TGCTTTATGGAAATAAACAGGTCA
BPKG47437	GTGACAAAAATGAACAAGAGGAAT	GTGACAAAAATGAACAAGAGGAAT
Gene_12607_1/1	AGAAACATTCTAGTCGGCGATAAC	AGAAACATTCTAGTCGGCGATAAC
isotig25105	AGAAATGATCACATCGACGACTAA	AGAAATGATCACATCGACGACTAA
isotig26419	ACACTCAATCTGGTAGATCACGAA	ACACTCAATCTGGTAGATCACGAA
PDZRN4	TGTGGAGATCTGTTAAAAATCGAA	TGTGGAGATCTGTTAAAAATCGAA
TNS1	ATATTCAGGAGCTTATGGACTTGC	ATATTCAGGAGCTTATGGACTTGC
znf840	AATATTTCGCTAAATGGCCTACAG	AATATTTCGCTAAATGGCCTACAG
